# SIRT1/P53 in retinal pigment epithelial cells in diabetic retinopathy: a gene co-expression analysis and He-Ying-Qing-Re formula treatment

**DOI:** 10.3389/fmolb.2024.1366020

**Published:** 2024-04-03

**Authors:** Shuyan Zhang, Jiajun Wu, Leilei Wang, Lin Mu, Xiaoyu Xu, Jiahui Li, Guoyi Tang, Guang Chen, Cheng Zhang, Yinjian Zhang, Yibin Feng

**Affiliations:** ^1^ Department of Ophthalmology, Longhua Hospital, Shanghai University of Traditional Chinese Medicine, Shanghai, China; ^2^ School of Chinese Medicine, Li Ka Shing Faculty of Medicine, The University of Hong Kong, Pok Fu Lam, Hong Kong SAR, China; ^3^ Shanghai Eye Disease Prevention and Treatment Center, Shanghai, China; ^4^ Eye, Ear, Nose and Throat Hospital, Fudan University, Shanghai, China

**Keywords:** diabetic retinopathy, Chinese herbal formula, Sirt1/p53, retinal pigment epithelial cells, weighted gene co-expression network analysis

## Abstract

**Objective:**

Diabetic retinopathy (DR) is a severe diabetic complication that leads to severe visual impairment or blindness. He-Ying-Qing-Re formula (HF), a traditional Chinese medicinal concoction, has been identified as an efficient therapy for DR with retinal vascular dysfunction for decades and has been experimentally reported to ameliorate retinal conditions in diabetic mice. This study endeavors to explore the therapeutic potential of HF with key ingredients in DR and its underlying novel mechanisms.

**Methods:**

Co-expression gene modules and hub genes were calculated by weighted gene co-expression network analysis (WGCNA) based on transcriptome sequencing data from high-glucose-treated adult retinal pigment epithelial cell line-19 (ARPE-19). The chromatographic fingerprint of HF was established by ultra-performance liquid chromatography coupled with high-resolution mass spectrometry (UPLC-Q-TOF-MS). The molecular affinity of the herbal compound was measured by molecular docking. Reactive oxygen species (ROS) was measured by a DCFDA/H2DCFDA assay. Apoptosis was detected using the TUNEL Assay Kit, while ELISA, Western blot, and real-time quantitative reverse transcription polymerase chain reaction (qRT-PCR) were used for detecting the cytokine, protein, and mRNA expressions, respectively.

**Results:**

Key compounds in HF were identified as luteolin, paeoniflorin, and nobiletin. For WGCNA, ME-salmon (“protein deacetylation”) was negatively correlated with ME-purple (“oxidative impairment”) in high-glucose-treated ARPE-19. Luteolin has a high affinity for SIRT1 and P53, as indicated by molecular docking. Luteolin has a hypoglycemic effect on type I diabetic mice. Moreover, HF and luteolin suppress oxidative stress production (ROS and MDA), inflammatory factor expression (IL-6, TNF-α, IL1-β, and MCP-1), and apoptosis, as shown in the *in vivo* and *in vitro* experiments. Concurrently, treatment with HF and luteolin led to an upregulation of SIRT1 and a corresponding downregulation of P53.

**Conclusion:**

Using HF and its active compound luteolin as therapeutic agents offers a promising approach to diabetic retinopathy treatment. It primarily suppressed protein acetylation and oxidative stress via the SIRT1/P53 pathway in retinal pigment epithelial cells.

## 1 Introduction

Diabetic retinopathy (DR) is one of the critical contributors to vision loss among diabetic patients. The International Diabetes Federation (IDF) reported in 2022 that approximately 537 million adults worldwide are affected by diabetes. DR patients are expected to increase to 643 million by 2030 and 783 million by 2045 (Jiang et al., 2023a). Due to its insidious onset, many patients are only diagnosed at advanced, often proliferative stages, highlighting the urgency of developing more effective treatment strategies. The pathology of DR is intrinsically linked with the degeneration of retinal pigment epithelial (RPE) cells, which are vital for ocular health and undergo substantial changes under diabetic conditions. It is characterized by increased inflammation, oxidative stress, and cell death. The degeneration of RPE cells is strongly associated with diabetic macular edema (Dolinko et al., 2020; Zhang J. et al., 2022). The alteration of these cells plays a significant role in the progression of DR, underscoring the need for targeted therapeutic approaches. Current treatments for DR have numerous challenges, including high costs, prolonged treatment durations, and poor patient adherence. These limitations necessitate the exploration of more effective, accessible, and patient-friendly treatment modalities for DR.

**FIGURE 1:**
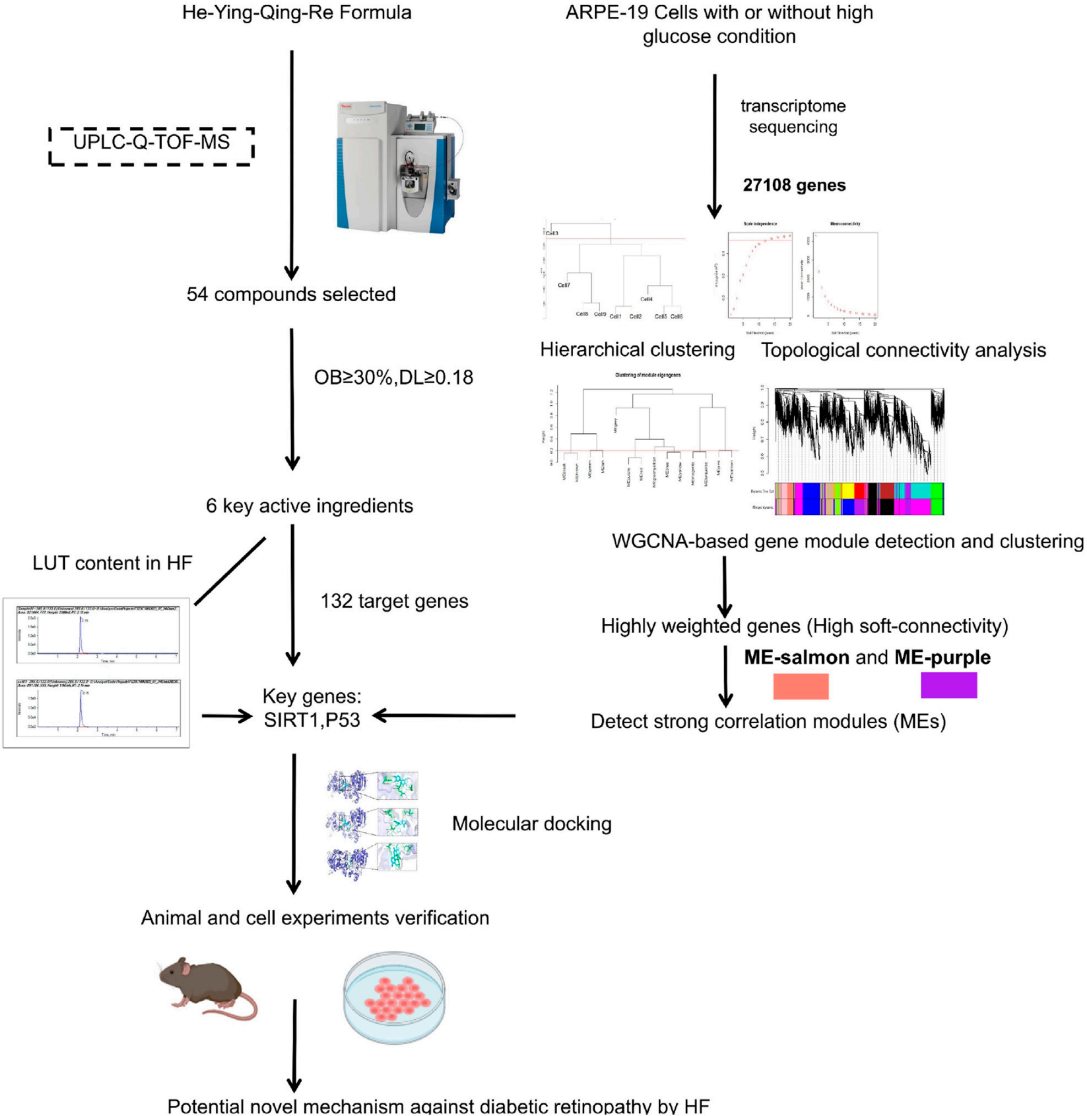
Flow chart of the study. First, the chromatographic fingerprint of HF was established by ultra-performance liquid chromatography coupled with high-resolution mass spectrometry. Meanwhile, co-expression gene modules and hub genes were calculated by WGCNA based on transcriptome sequencing data from high-glucose-treated adult retinal pigment epithelial cell line-19. Then, herbal compound molecular affinity was measured by molecular docking, and *in vitro* and *in vivo* experimental validation was conducted.

In this challenging landscape, the He-Ying-Qing-Re formula (HF), a traditional Chinese medicinal concoction, emerges as a promising alternative. HF was formed based on “Si-Miao-Yong-An decoction” (SM), established in the Qing Dynasty. Growing contemporary evidence has demonstrated that SM is beneficial in improving blood circulation, reducing inflammation and immune responses, and promoting angiogenesis (Li et al., 2020; Du A et al., 2021). SM could also attenuate DR development by abrogating the expression of NF-κB, TNF-α, IL-6, IL-8, and MMP9 (Chen et al., 2021; Cui et al., 2023). Clinically, HF has shown promising therapeutic effects on DR for decades (Ping, 2007a; Ping, 2007b). For instance, Zhang (2012) found that combined HF and laser therapy can alleviate clinical symptoms and improve eyesight and fundus vascular conditions effectively. Using a streptozotocin (STZ)-induced DR mouse model, we found that HF could inhibit advanced glycation end products (AGEs) and ameliorate endothelial dysfunction via modulating tight junctions and AGEs downstream signaling against DR (Wang et al., 2015). Furthermore, using the STZ-induced DR mouse model, we found that HF improves retinal neurodegeneration by blocking endoplasmic reticulum and mitochondria-dependent oxidative stress (Zhang et al., 2018). Considering the protective effects of HF on endothelial cells and neuronal cells identified in the STZ-induced mouse model, we consider that HF may also confer protection to other retinal cells by reversing the pathological condition of DR, which can also be validated in the STZ-induced mouse model. Since HF is shown to restore the breakdown of the blood–retinal barrier in DR mice in these two previous studies (Wang et al., 2015; Zhang et al., 2018), we hypothesized the potential protective effect of HF on retinal pigment epithelial cells in DR due to its key role in maintaining retinal function and integrity. The dysfunction of retinal pigment epithelial cells may contribute to the breakdown of the blood–retinal barrier in DR mice, while it may be abolished by HF treatment. Our study will address the comprehensive therapeutic effects of HF on multiple facets of DR pathology. The study design includes a combination of weighted gene co-expression network analysis (WGCNA)-related computational analysis and *in vitro* and *in vivo* experimental validation (as shown in Figure 1).

A critical aspect of our research is the novel mechanism of the SIRT1/P53 pathway involved in the molecular pathogenesis of DR. It is a breakthrough that extends our understanding to both cellular and animal levels. This discovery is a significant advancement in the field of diabetic retinopathy research. Traditionally, the SIRT1/P53 pathway is recognized for its role in regulating cellular longevity, DNA repair, and apoptosis (Katayoshi et al., 2021; Yan et al., 2021; Dong et al., 2023; Xu et al., 2023). The existing literature predominantly links this pathway to the modulation of age-related and carcinogenic processes, where SIRT1 acts as a deacetylase to stabilize P53 and regulate its activity. However, our findings diverge from these conventional roles, uncovering a unique interaction within the DR context. In our study, the SIRT1/P53 pathway emerges as a central player in managing crucial cellular functions, specifically protein acetylation and oxidative stress, both of which are fundamental in the progression of DR. This novel mechanism extends beyond the established understanding of SIRT1/P53, presenting a more comprehensive perspective. It highlights an intricate balance in cellular processes, thereby offering a substantial expansion to the current knowledge and paving the way for new avenues for targeted therapeutic strategies for DR.

## 2 Materials and methods

### 2.1 Preparation of lyophilized powder of HF

As shown in Table 1, HF contains 11 herbs, and all the plant names have been certified (www.theplantlist.org). Raw herbs were purchased from Longhua Hospital, affiliated with the Shanghai University of Traditional Chinese Medicine. Different grams of raw herbs were weighed, and 10 times the volume of ddH_2_O (w/v) was added to cover the mixtures. The herbs were left to soak for 30 min and then boiled twice for up to an hour each time. The sample solution was then filtered by filtration. We concentrated the herbal liquid to a certain volume and then freeze-dried it to obtain HF lyophilized powder.

**TABLE 1:** Ingredient information in HF.

Botanical name	Herbal name	Chinese name	Voucher no	LOT
*Lonicera japonica Thunb*	Flos Lonicerae	Jin-Yin-Hua	TS23C100-002	No. 230130
*Angelica sinensis (Oliv.) Diels*	Radix Angelicae Sinensis	Dang-Gui	TS23C100-009	No. 230208
*Scrophularia ningpoensis Hemsl*	Radix Scrophulariae	Xuan-Shen	TS23C100-010	No. 23013103
*Rehmannia glutinosa (Gaertn.) Libosch. exFisch. and C.A. Mey*	Radix Rehmanniae Exsiccata	Sheng-Di	TS23C100-012	No. 230306
*Lycium barbarum L*	Fructus Lycii Barbari	Gou-Qi-Zi	TS23C100-005	No. 230417–1
*Atractylodes macrocephala Koidz*	Rhizoma Atractylodis Macrocephalae	Bai-Zhu	TS23C100-003	No. 230302–1
*Cornus officinalis Sieb.et Zucc*	Fructus Corni	Shan-Zhu-Yu	TS23C100-004	No. 230309
*Paeonia suffruticosa Andr*	Cortex Moutan	Mu-Dan-Pi	TS23C100-006	No. 202321
*Cuscuta chinensis Lam*	Semen Cuscutae	Tu-Si-Zi	TS23C100-007	No. 230424
*Ligustrum lucidum Ait*	Fructus Ligustri lucidi	Nv-Zhen-Zi	TS23C100-008	No. 230410
*Citrus reticulata Blanco*	Pericarpium Citri Reticulatae	Chen-Pi	TS23C100-011	No. 230420

### 2.2 Chemical composition analysis

UPLC-Q-TOF/MS was used to analyze HF and luteolin (Sigma, USA). The chemical composition and chromatographic and mass spectrometry conditions are mentioned in Supplementary Material S1. Analyst TF 1.7.1 and PeakView 1.2 were used for data acquisition and processing separately. Identification was prioritized by matching the mass spectrometry data with the Natural Products HR-MS/MS Spectral Library 1.0 database.

### 2.3 Optimal drug concentration screening

ARPE-19 cells were purchased from the Cell Bank of the Chinese Academy of Sciences. The cells were cultured at 37°C in an incubator with 5% CO_2_ and were used for experiments at passages 3 to 7. Intervention with ARPE-19 cells in high-glucose conditions (5.5, 15, 25, 35, 45, 55, and 65 mM, Sigma, USA) was carried out for 24 h and 48 h to mimic DR *in vivo*. The Cell Counting Kit-8 (CCK-8) (APExBIO, USA) was used to calculate the concentration and treatment time when cell viability was reduced to approximately 50%.

### 2.4 RNA isolation and library preparation

Total RNA was extracted using the TRIzol reagent (Invitrogen, CA, United States) according to the manufacturer’s protocol. RNA purity and quantification were evaluated using the NanoDrop 2000 Spectrophotometer (Thermo Fisher Scientific, United States). RNA integrity was assessed using the Agilent 2100 Bioanalyzer (Agilent Technologies, Santa Clara, CA, United States). Then, the libraries were constructed using the VAHTS Universal V6 RNA-seq Library Prep Kit according to the manufacturer’s instructions. The transcriptome sequencing and analysis were conducted by OE Biotech Co., Ltd. (Shanghai, China).

### 2.5 RNA sequencing and differentially expressed gene analysis

The libraries were sequenced on the Illumina NovaSeq 6000 platform, and 150 bp paired-end reads were generated. Approximately 50.42 raw reads for each sample were generated. Raw reads in FASTQ format were first processed using fastp, and low-quality reads were removed to obtain clean reads. Then, approximately 48.15 clean reads for each sample were retained for subsequent analyses. The clean reads were mapped to the reference genome using HISAT2. The FPKM of each gene was calculated, and the read counts of each gene were obtained by HTSeq-count.

### 2.6 Weighted gene co-expression network analysis

Filtered transcriptomic data (10,843 genes) were used to construct a sample hierarchical cluster tree (Supplementary Material S2), in which differently colored branches represent different gene modules (cutHeight = 275,000 and minSize = 2). To satisfy the correlation between genes conforming to the scale-free topological distribution, the soft threshold β was adopted as the value with the smallest β value when the scale-free topological fit index >0.8 or when a plateau period was reached. Then, clustering was performed using the dissTOM of dissimilarity. “MinModulesSize” and “MedissThres” were set to 340 and 0.2, respectively, while the module serial number was converted to color, and the module eigengene (ME) was calculated and clustered by its dissimilarity. Eventually, we identified highly correlated modules and hub genes based on Pearson’s r, *p*-value, functional annotation, and biological phenotype analysis (Supplementary Material S3).

### 2.7 Molecular docking

SIRT1 (code: 4I5I) and P53 (code: 6GGE) 3D structures were downloaded from the PDB database (https://www.rcsb.org), and water molecules and small-molecule ligands were removed using Pymol 2.5.5 (https://www.pymol.org). Luteolin (CID: 5280445), paeoniflorin (CID: 442534), and nobiletin (CID: 72344) 3D structures were obtained from the PubChem compound database and used for ligand docking. Hydrogenation was performed by AutoDockTools, and the active pocket position was set (https://autodocksuite.scripps.edu/adt/). All molecules were docked using AutoDock Vina 1.1.2, and the binding energy was subsequently calculated.

### 2.8 Induction of DR in mice and treatment

Animal experiments were approved by Beijing Viton Lever Experimental Technology Co. Ltd. (PA23060701). A stable diabetes model can be constructed using male mice (Polewik et al., 2023). Eight-week-old male C57BL/6 mice (25 ± 2 g) were purchased from the Animal Research Center of Viton Lever Experimental Technology Co., Ltd. (Shanghai, China). The mice were acclimatized and fed for 1 week to start the experiments at the animal research center. After continuous intraperitoneal injection of STZ solution (55 mg/kg) for 5 days, blood glucose levels were tested for 1 week. Mice with blood glucose levels higher than 15 mmol/L on three consecutive occasions were included in the study. The mortality of modeling was 1%.

Then, STZ-induced mice were segmented into four groups randomly. Primarily, untreated diabetic mice (n = 5) were regarded as the model group. Afterward, the remaining mice with hyperglycemia were administered three doses of HF (50 mg/kg (low dose; n = 5), 75 mg/kg (middle dose; n = 5), and 100 mg/kg (high dose; n = 5) and luteolin (20 mg/kg; n = 5) via daily gavage, respectively. Untreated non-diabetic mice (n = 5) were categorized as the normal control. The control and model groups were given ddH_2_O daily. At the end of the study, the mice were anesthetized using isoflurane delivered via a vaporizer set to 4% for induction. Once fully anesthetized, confirmed by lack of pedal reflex, euthanasia was conducted using an overdose of pentobarbital (200 mg/kg) by intraperitoneal injection to perform humane death. This method was chosen for its efficacy and minimal stress on the animals.

### 2.9 Measurement of RBG, IPGTT, and weight

To evaluate the hypoglycemic influence of HF and luteolin, we measured random blood glucose (RBG) levels and conducted an intraperitoneal glucose tolerance test (IPGTT) using a glucometer (Bayer, Germany). Weight and RBG assessments were performed every 2 weeks when the blood glucose levels remained higher than 15 mmol/L. IPGTT was followed by measurements at 0.5, 1, 1.5, and 2 h after injecting blood glucose (2 g/kg). The glucose tolerance capacity of the mice was assessed by calculating the area under the curve (AUC) and plotting it according to relevant 5-time points in each group.

### 2.10 Cell culture and treatment

The CCK-8 assay was used to detect HF (0, 5, 10, 20, 40, 80, 160, 320, and 640 μg/mL) and luteolin (0, 10, 20, 40, 60, and 80 μM) toxicity. Absorbance optical density (AOD) was measured at 450 nm using a microplate reader (BioTek, USA). Eventually, cells were grown in a medium with an optimal high glucose concentration (35 mM) for 48 h and then treated with different doses of HF (40, 80, and 160 μg/mL) for 48 h or luteolin (40 μM) for 24 h.

### 2.11 Enzyme-linked immunosorbent assay

Enzyme-linked immunosorbent assay (ELISA) was used to calculate the anti-oxidative stress and anti-inflammatory values of HF and luteolin *in vivo* and *in vitro* by the corresponding kit: malondialdehyde (A003-1-2, Nanjing Jiancheng Bioengineering Institute, China), IL-6 (MM-0163M2, MM-0149H2), MCP-1 (MM-46708M2), TNF-α (MM-0132M2, MM-0122H2), and IL1-β (MM-0181H2, Mmbio, China).

### 2.12 Retina and ARPE-19 cell apoptosis

Retina and ARPE-19 cell apoptosis levels were detected using the One-Step TUNEL Apoptosis Assay Kit, according to the procedures from the manufacturer (C1089, Beyotime, China). According to the manufacturer’s instructions, paraffin-embedded eyeballs were dewaxed in xylene (5–10 min). Next, xylene was replaced, and dewaxing was performed for 5–10 min in the following sequence: anhydrous ethanol (5 min), 90% ethanol (2 min), 70% ethanol (2 min), and distilled water (2 min). The cells were fixed with 4% paraformaldehyde (G1101, Servicebio, China) for 30 min. Then, 20 μg/mL of protease K without DNase (ST532, Beyotime, China), immunostaining washing solution (P0106, Beyotime, China), and 50 μL of TUNEL test solution were added for 60 min in the dark.

### 2.13 ROS assay

The DCFDA/H2DCFDA-Cellular ROS Assay Kit (Ab113851, Abcam, UA) was used to detect ROS in ARPE-19 cells. The cells in all groups were washed with 1×Buffer, and then DCFDA solution was added and kept for 45 min at 37°C in the dark. Finally, the ROS fluorescence intensity was observed under an inverted fluorescence microscope (Nikon, Japan).

### 2.14 Immunohistochemistry and immunofluorescence staining

According to the manufacturer’s procedures, we performed immunohistochemistry in paraffin-embedded eyeballs and immunofluorescence staining in ARPE-19 cells. Next, SirT1 mouse mAb (1F3, Cst, USA, 1:100) and anti-p53 antibody (PAb240, Abcam, United Kingdom, 1:500) were incubated at 4°C overnight. Antibody dilution buffer (1× PBS/1% BSA/0.3% Triton™ X-100) was used to dilute the fluorescent substance-labeled secondary antibody (ZF-0317, Zsgb-Bio, China, 1:400). Then, retinal tissue and cells were incubated for 1–2 h and protected from light. Immunofluorescence images were acquired using an inverted fluorescence microscope (ECLIPSE Ti2, Nikon, Japan).

### 2.15 Western blotting

Retinal tissue and ARPE-19 cells in each group were lysed using RIPA lysis buffer (P0013B, Beyotime, China), supplemented with protease inhibitor (410090, Bimake, United States) and phosphatase inhibitor (510042, Bimake, United States) on ice. The liquid supernatant was analyzed using the BCA method (P0010, Beyotime, China), followed by electrophoresis, membrane transfer (PVDF, Bio-Rad), and antibody incubation ((SirT1 mouse mAb, 1F3, Cst, USA), (anti-p53 antibody, PAb240, Abcam, United Kingdom), (acetyl-p53 antibody, Lys382, Cst, United States), (antibodies against acetyl-Lysine, #9441, Cst, United States), and (GAPDHXP® Rabbit mAb) (D16H11, Cst, United States)) overnight at 4°C. After that, secondary antibodies ((anti-rabbit IgG, HRP-linked antibody, #7074, Cst, USA) and (rhodamine-labeled rabbit anti-goat IgG, ZF-0317, Zsgb-Bio, China)) were added and gently shaken at room temperature for 2 h. Protein samples were visualized using an ECL chemiluminescence kit (A + B, Bio-Rad, USA) (Supplementary Material S3).

### 2.16 Quantitative real-time PCR

RNA was extracted from ARPE-19 cells by TRIzol lysis (R0016, Beyotime, China), reverse transcribed into cDNA, and mRNA expression of SIRT1 and TP53 genes was detected by real-time PCR. The primers used were as follows: GAPDH: AAA​ATC​AAG​TGG​GGC​GAT​GC (forward), TGG​TTC​ACA​CCC​ATG​ACG​AA (reverse); SIRT1: TGT​GTC​ATA​GGT​TAG​GTG​GTG​A (forward), AGC​CAA​TTC​TTT​TTG​TGT​TCG​TG (reverse); and P53: CAG​CAC​ATG​ACG​GAG​GTT​GT (forward), TCA​TCC​AAA​TAC​TCC​ACA​CGC (reverse). The relative gene expression was calculated using the comparative CT method.

### 2.17 Statistical analysis

GraphPad Prism version 7.0 software was used for statistical analysis. All values were expressed as the mean ± SD. *p < 0.05, **p < 0.01, and ***p < 0.001 were considered statistically significant. A one-way analysis of variance (ANOVA) was used for comparison among multiple groups.

## 3 Results

### 3.1 Chromatographic fingerprinting and target prediction of HF

**FIGURE 2:**
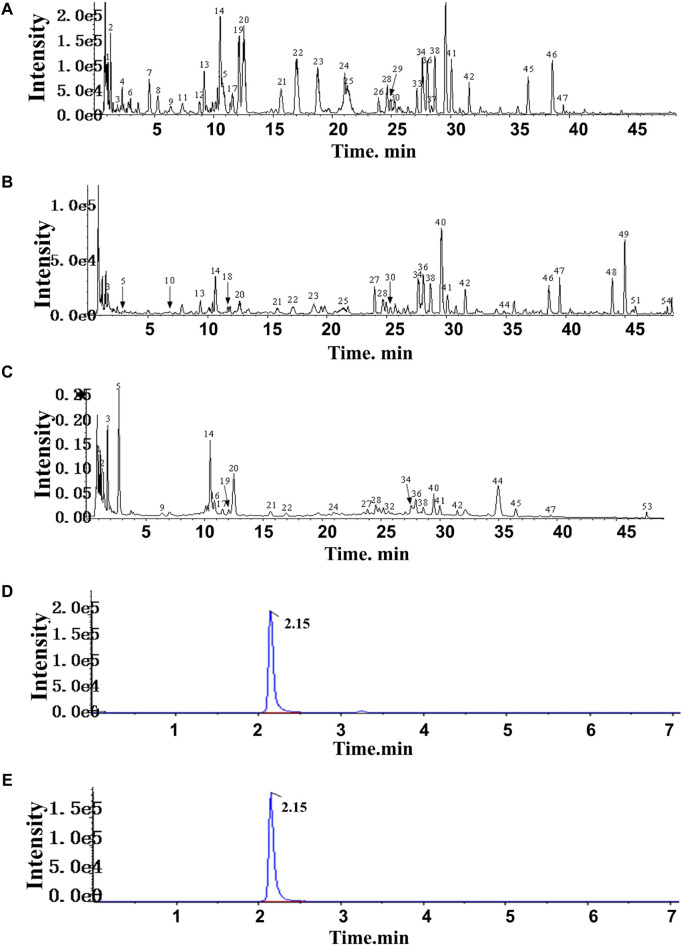
TIC of He-Ying-Qing-Re formula. **(A)** Positive ion mode. **(B)** Negative ion mode. **(C)** UPLC-UV chromatogram. **(D)** Chromatogram of the sample of He-Ying-Qing-Re formula. **(E)** Chromatogram of luteolin.

In HF, 54 compounds were identified from the extracts by UPLC-HRMS. A total of 45/54 compounds were used for further target prediction and validation (Supplementary Material S4). Figure 2 shows the UPLC-HRMS base peak ion flow pattern in both positive and negative ion modes, as well as the UPLC-UV chromatograms of HF. Based on the criteria of “oral bioavailability (OB) ≥ 30% and drug-like properties (DL) ≥ 0.18”, six drug-active ingredients were filtered out (Table 2). Moreover, 132 targets of action corresponding to the active ingredients were retrieved from databases (Supplementary Material S5). The databases include TCMSP (http://tcmspw.com/tcmsp.php) and HERBS (http://herb.ac.cn). The gene abbreviations of the target proteins were determined by the UniProt database (http://www.unitprot.org/).

**TABLE 2:** Basic information on the active ingredients of HF.

Number	Chemical compound	Mol ID	OB(%)	DL (%)
1	Nobiletin	MOL005828	61.67	0.52
2	Paeoniflorin	MOL001924	53.87	0.79
3	Luteolin	MOL000006	36.16	0.25
4	Atractylenolide Ⅲ	MOL000072	35.95	0.21
5	Aucubin	MOL002813	35.56	0.33
6	Benzoylpaeoniflorin	MOL007025	31.14	0.54

### 3.2 Identification of hub genes in ARPE-19 cells under high-glucose conditions

**FIGURE 3:**
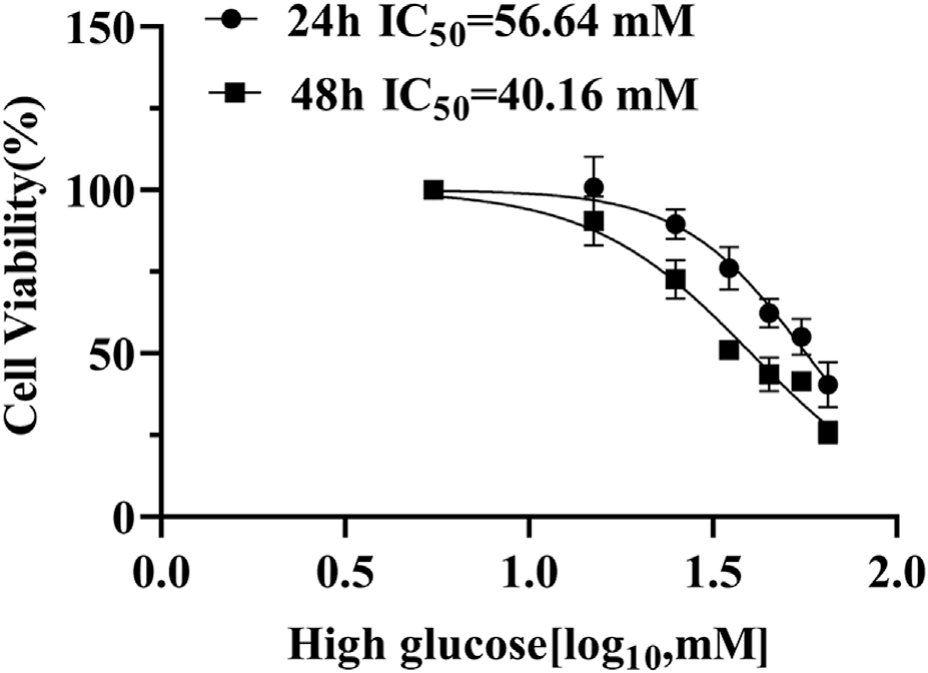
ARPE-19 cell viability cultivated with high glucose for 24 h and 48 h.

To find the optimal high-glucose concentration for ARPE-19 cell transcriptome sequencing, we performed the CCK-8 assay first. High-glucose-induced cell viability was significantly inhibited in a concentration-dependent manner. Notably, we chose high glucose with 35 mM for 48 h, when viability decreased to approximately 50% (Figure 3).

#### 3.2.1 Constructing weighted co-expression gene modules

**FIGURE 4:**
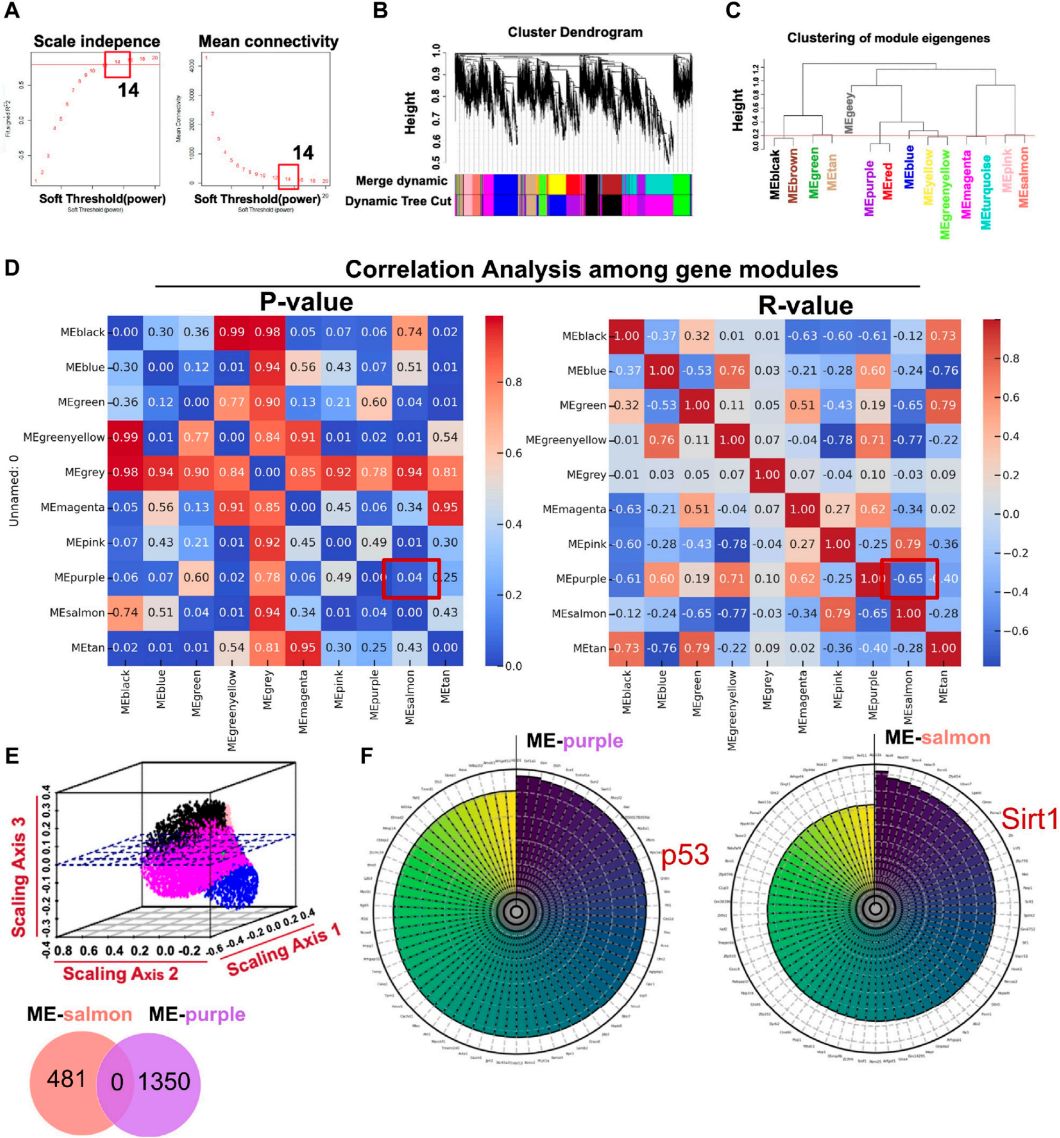
Core modules calculated by WGCNA with transcriptome sequencing data associated with ARPE-19 cells treated in a high-glucose condition (35 mM). **(A)** Analysis of network topology for powers of soft threshold. **(B)** Gene clustering dendrogram containing modules in hierarchical clustering and a heatmap. **(C)** Highly relevant gene modules in accordance with the value of the module eigengene (cut-off height = 0.2). **(D)** Pearson’s r and *p*-value between modules (*p* < 0.05; *R* ^2^ = −0.6542 between ME-salmon and ME-purple). **(E)** The upper panel is a geometric interpretation of gene expression in a 3D scatter plot. The relevant genes between the modules are shown in the nether Venn diagram. **(F)** Top 70 hub genes in the modules ranked by soft-connectivity (ME-salmon and ME-purple).

Using the R package WGCNA, we analyzed transcriptome sequencing data from ARPE-19 cells treated in high-glucose conditions. Expression variances greater than the 60th percentile of the whole genome (10,843 genes) were selected in hierarchical clustering. The scale-free soft threshold (14) was determined by scale independence and mean connectivity (Figure 4A). The co-expressed gene modules are shown in Figure 4B. Eventually, 13 co-expression modules were clustered into 10 modules (Figure 4C). Figure 4D shows that ME-salmon was strongly linked with ME-purple, but it exhibits a negatively correlated regulatory pattern (*p* < 0.05; *R* ^2^ = −0.6542). The 3D scatter plot (Figure 4E) and Venn diagram (upper panel) illustrate that genes between the two modules were independent of each other. Highly correlated key genes (Top 70) in the salmon and purple modules were identified through intramodular soft-connectivity analysis (Figure 4F).

#### 3.2.2 GO and KEGG analysis of highly relevant modules

**FIGURE 5:**
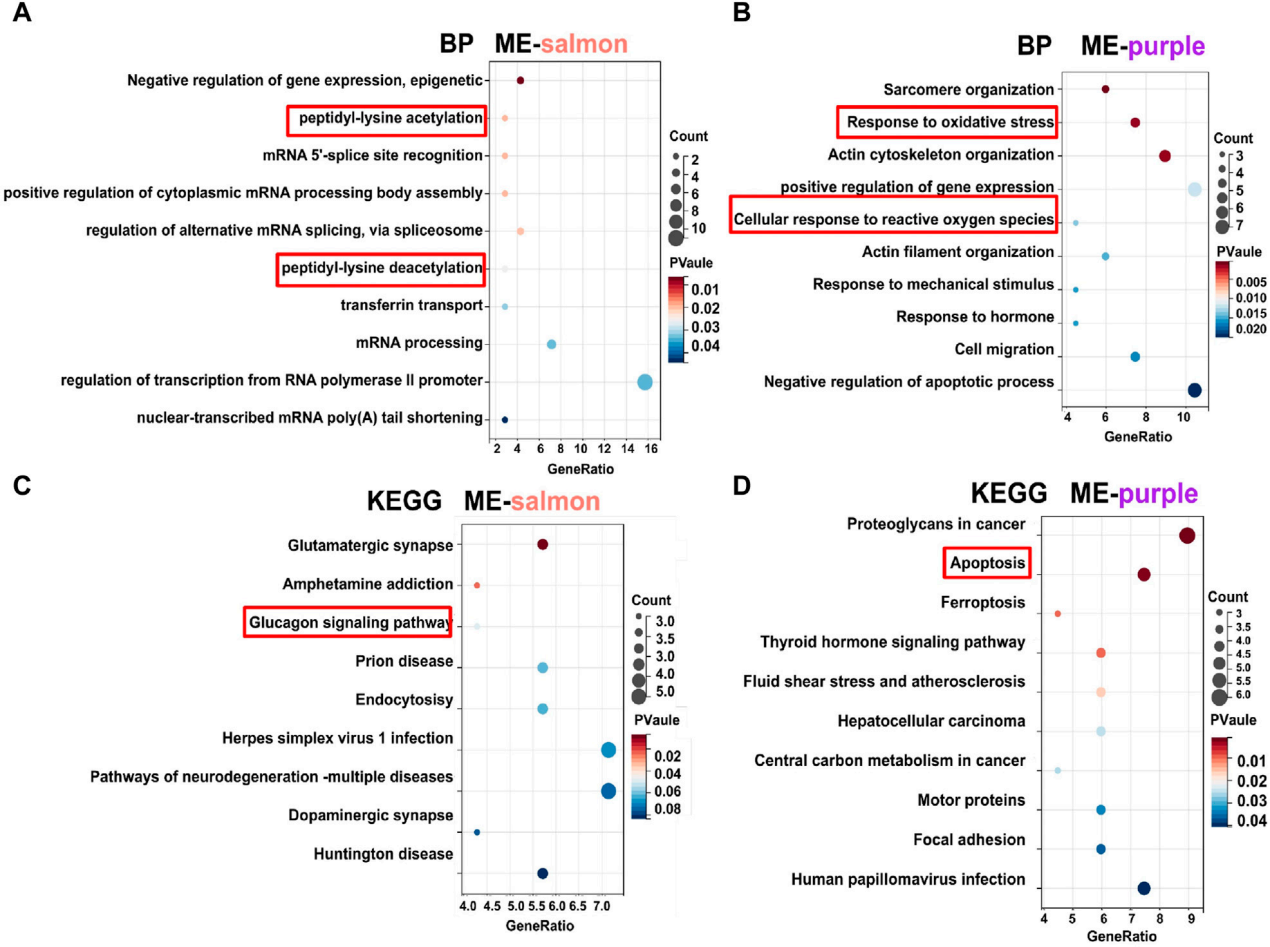
GO and KEGG analysis of gene modules. **(A, C)** Functional enrichment analysis for genes (top 70) in the ME-salmon module. **(B, D)** Functional enrichment analysis for genes (top 70) in the ME-purple module.

In terms of functional analysis among ME-salmon and ME-purple modules, ME-salmon was chiefly enriched in the Gene Ontology (GO) terms: “0018394: peptidyl-lysine acetylation (*p* = 0.019),” “0034983: peptidyl-lysine deacetylation (*p* = 0.025),” and “0045814: negative regulation of gene expression, epigenetic (*p* = 0.006).” Additionally, it was enriched in the KEGG-term “hsa04922: glucagon signaling pathway (*p* = 0.049),” which is shown in Figures 5A, C. Significant epigenetic changes included DNA methylation, histone modifications (protein acetylation), and noncoding RNAs, which implied promising perspectives defeating DR (Suarez et al., 2023). Meanwhile, DR is a retina neurodegenerative disease, and relevant studies have reported that high-glucose levels promoting inflammatory factors release through protein acetylation (Wu et al., 2022). Thus, ME-salmon may be classified as “protein deacetylation.” In Figures 5B, D, ME-purple were prevailingly concentrated in GO “0006979: response to oxidative stress (*p* = 0.001),” “0034614: cellular response to reactive oxygen species (*p* = 0.014),” and “0042981: regulation of apoptotic process (*p* = 0.035).” It was enriched in the KEGG-term “hsa04210: apoptosis (*p* = 0.001).” Oxidative stress has been shown a key factor in DR pathogenesis. Excessive ROS accumulation induces mitochondrial damage, apoptosis, inflammation, and lipid peroxidation, leading to structural and functional damage to the retina. Herein, ME-purple may represent “oxidative impairment.” In conclusion, regulating “protein deacetylation” and “oxidative impairment” would be a new therapeutic approach against DR.

In Figure 4F, we demonstrated the highly associated genes (top 70) in ME-salmon and ME-purple. Subsequently, hub genes were integrated into two modules with core targets in HF. Finally, SIRT1 and P53 were singled out. The finding indicated they may function together to influence DR through molecular pathways and genetic networks within their respective modules.

### 3.3 Molecular docking of core targets and active compounds

**FIGURE 6:**
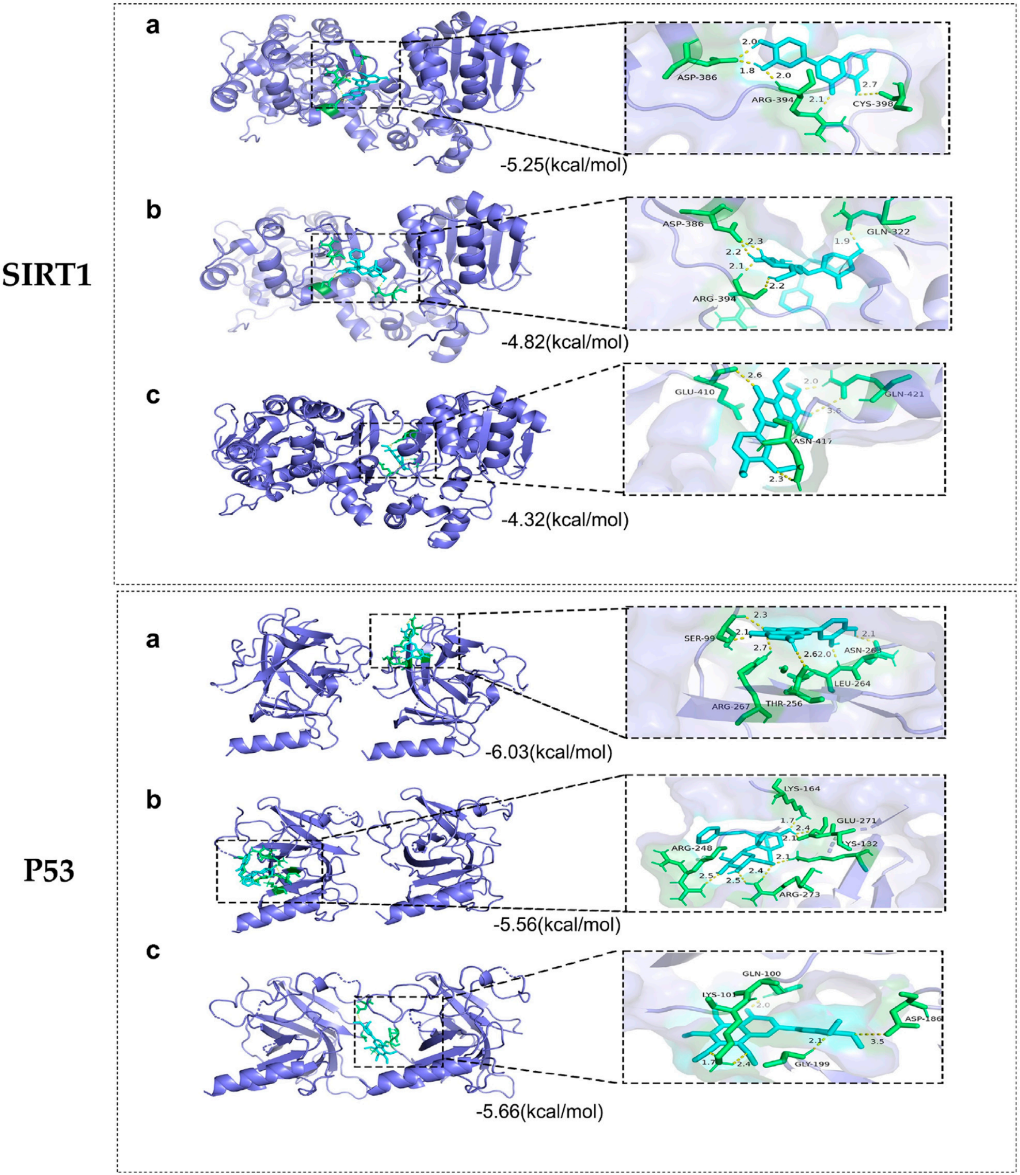
Molecular docking of core targets with compounds. SIRT1 docking with **(A)** luteolin, **(B)** paeoniflorin, and **(C)** nobiletin. P53 docking with **(A)** luteolin, **(B)** paeoniflorin, and **(C)** nobiletin.

Whether the active compounds can directly act on SIRT1 and P53 is still unrevealed; thus, molecular docking was performed to deepen comprehension in repairing visual impairment. We chose the top three compounds (nobiletin, paeoniflorin, and luteolin) to dock with SIRT1 and P53. According to the VINA junction auto-scoring, the binding energies were all less than −4 kcal/mol (Table 3), indicating that the ligand and receptor can bind spontaneously. Figure 6 shows that luteolin is bound to ASP-386, ARG-394, and CYS-398 of SIRT1, increasing structural stability. Paeoniflorin formed an indestructible space with SIRT1 at GLN-322, ASP-386, and ARG-394. Similarly, nobiletin constructed a tight connection with SIRT1 at the catalytic sites of GLU-410, ASN-417, and GLN-421. Luteolin is embedded in the protein cavity of P53 to set up an impregnable structure with SER-99, ASN-263, LEU-264, ARG-267, and THR-256. Simultaneously, paeoniflorin interacted with P53 at the binding sites of ARG-273, GLU-271, LYS-164, ARG-248, and YS-132. The hard connectivity of nobiletin binds with P53, owing to GLY-199, ASP-186, LYS-101, and GLN-100. In brief, luteolin seemed to bind tightly with SIRT1 and P53.

**TABLE 3:** Molecular docking of the active ingredients of HF with key targets of DR.

Target	Active ingredient	Docking energy (kcal/mol)	Numbers of H-Bonds	Amino acid residue
SIRT1	Luteolin	−5.25	5	ASP-386, ARG-394, and CYS-398
SIRT1	Paeoniflorin	−4.82	4	GLN-322, ASP-386, and ARG-394
SIRT1	Nobiletin	−4.32	5	GLU-410, ASN-417, and GLN-421
P53	Luteolin	−6.03	6	SER-99, ASN-263, LEU-264, ARG-267, and THR-256
P53	Paeoniflorin	−5.56	5	ARG-273, GLU-271, LYS-164, ARG-248, and YS-132
P53	Nobiletin	−5.66	6	GLY-199, ASP-186, LYS-101, and GLN-100

### 3.4 Determination of the luteolin content in HF

In addition, we evaluated the luteolin content (0.0004% with 0.6% RSD) in HF by HPLC-DAD (Figures 2C, D). This result implied that luteolin could be an alternative natural bioactive ingredient to improve retinal disorders generated by DR.

### 3.5 Therapeutic potential of HF and luteolin on STZ-induced diabetic mice

#### 3.5.1 Different performances in weight loss and hypoglycemic effect

**FIGURE 7:**
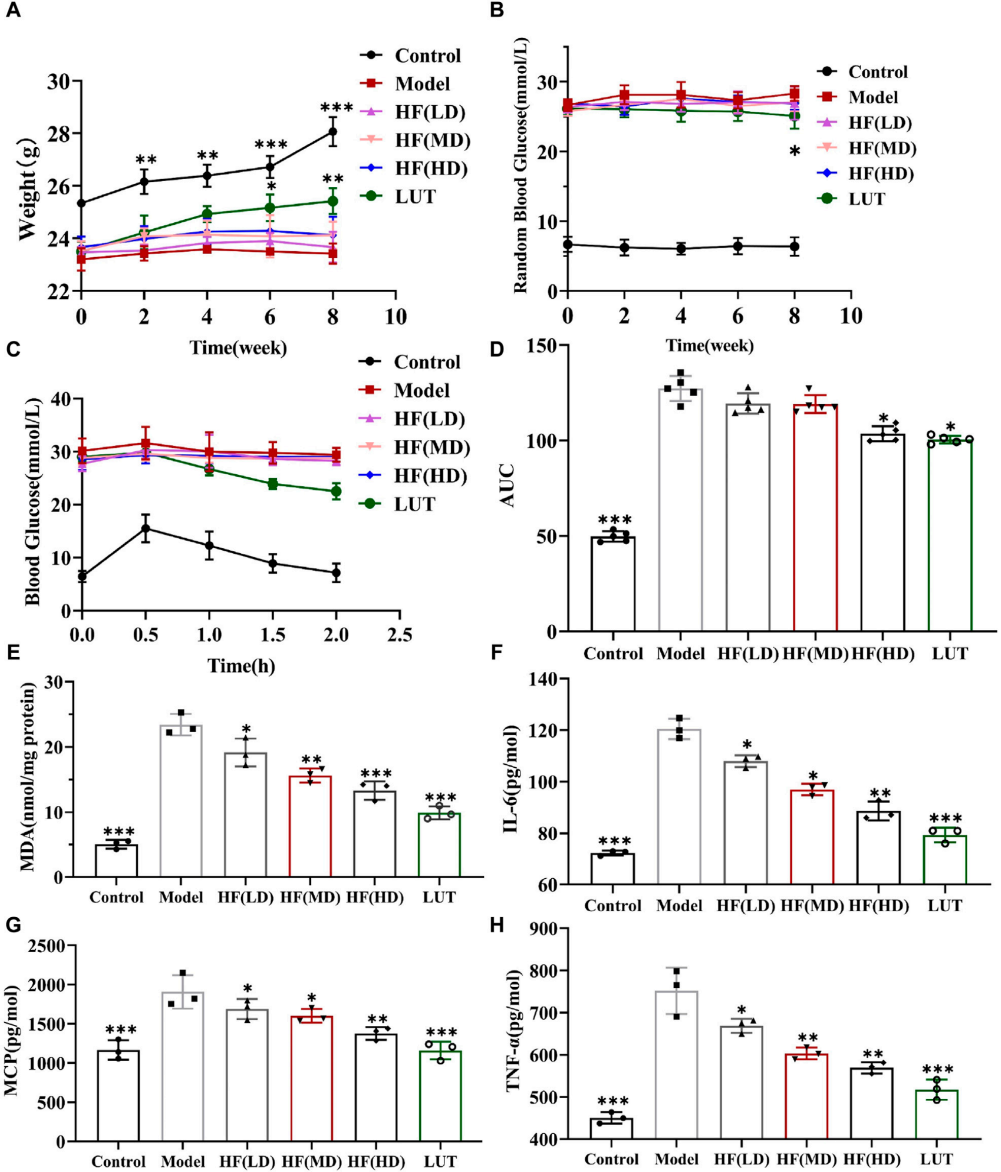
HF could not significantly decrease STZ-induced hyperglycemia along with weight loss but ameliorated oxidative stress and inflammatory response in diabetic mice. **(A)** Variation in body weight within various treatment methods. **(B)** Measurement of random blood glucose. **(C, D)** Intraperitoneal glucose tolerance test and its relevant areas under the curve (AUC) in diabetic mice. **(E–H)** Detecting the anti-oxidative stress (MDA) and anti-inflammatory (IL-6, MCP-1, and TNF-α) values of HF and luteolin in DR treatment by enzyme-linked immunosorbent assay. All data are presented as the mean ± SD. HF (LD): HF 50 mg/kg; HF (MD): HF 75 mg/kg; and HF (HD): HF 100 mg/kg *p < 0.05; **p < 0.01; and ***p < 0.001 *versus* model.

Hyperglycemia plays a fateful role in DR pathogenesis, inducing oxidative stress and inflammation. Thus, hypoglycemia seemed to be a potential therapeutic measure against DR. The current data on RBG, IPGTT, and AUC showed that luteolin could generate a slightly hypoglycemic effect on DR mice, with weight loss reversed in STZ-induced diabetic mice in later phases (Figures 7A–D, *p* > 0.05 vs. model). The finding was in accordance with relevant research reports (Alozieuwa et al., 2022; Ali et al., 2023). Nonetheless, as shown in Figures 7A–D, HF with different concentrations failed to reduce RBG (*p* all >0.05 vs. model). The low- and mid-dose HF treatment methods were weak in ameliorating glucose tolerance and hypoglycemic activity, along with weight loss. Luteolin possessed weight loss and a slight hypoglycemic effect on STZ-induced diabetic weight loss (*p* all >0.05 vs. model). With increasing concentrations, high-dose HF intervention improved glucose tolerance sensitivity (*p* < 0.05 vs. model), but there was no significant effect on weight loss control (*p* > 0.05 vs. model).

#### 3.5.2 Effective role in ameliorating inflammation and oxidative stress

MDA is a crucial parameter that reflects the potential antioxidant capacity and peroxidative damage degree. Meanwhile, IL-6, MCP-1, and TNF-α are inflammatory cytokines highly associated with DR development. Thus, we calculated those parameters’ expression by a corresponding kit to observe HF and luteolin protective effects on STZ-induced DR. In Figures 7E–H, MDA, IL-6, MCP-1, and TNF-α exhibited the peak values in the model group, whereas, treated with different doses of HF and luteolin, those cytokine expressions dramatically declined (*p* all >0.05 vs. model). In addition, luteolin showed stronger antioxidant stress and anti-inflammatory properties than HF (*p* all <0.05 vs. HF (LD, MD, and HD)). Regarding all the above-mentioned consequences, HF and luteolin may serve as promising treatments in DR progression.

#### 3.5.3 Improvement in apoptosis

**FIGURE 8:**
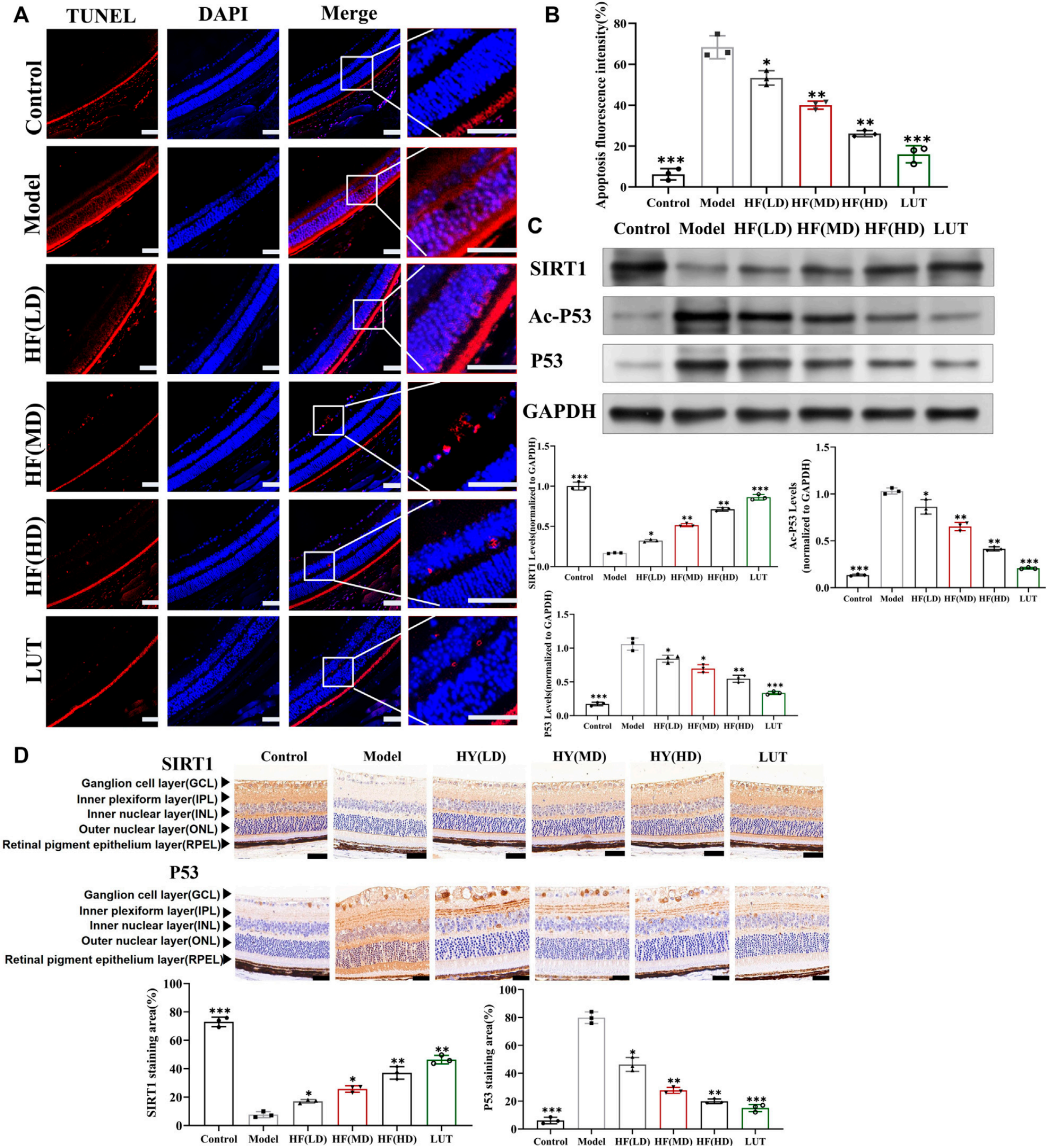
Result of the TUNEL Assay Kit used to observe anti-apoptosis with HF and luteolin treatment in diabetic mice. **(A)** The red color represents apoptotic cells. Typical performance is amplified. **(B)** TUNEL quantification analysis (n = 3). **(C)** Expression of SIRT1, Ac-P53, P53, and GAPDH at protein levels in the *in vivo* experiment (n = 3). **(D)** SIRT1 and p53 immunohistochemistry. Brown color indicates positive staining. HF(LD): HF 50 mg/kg; HF (MD): HF 75 mg/kg; and HF (HD): HF 100 mg/kg. Scale bar, 20 µm for all images. *p < 0.05; **p < 0.01; and ***p < 0.001 *versus* model.

As shown in Figure 8, retinal cells were a close-packed array, with appropriate cell numbers and extremely few apoptotic cells in the control group, while the model group had disorganized and sparse retinal cells, along with a significant increase in apoptotic cells. Low-dose HF was not as effective as middle- and high-dose treatment methods in preventing apoptosis. Interfered with luteolin, apoptotic cells exhibited a more drastic reduction than HF treatment. Therefore, we hypothesized that HF and luteolin played a crucial role in retina cell apoptosis against DR.

### 3.6 Protective effect of HF and luteolin in ARPE-19 cells under high-glucose conditions

#### 3.6.1 Increase in cellular viability

**FIGURE 9:**
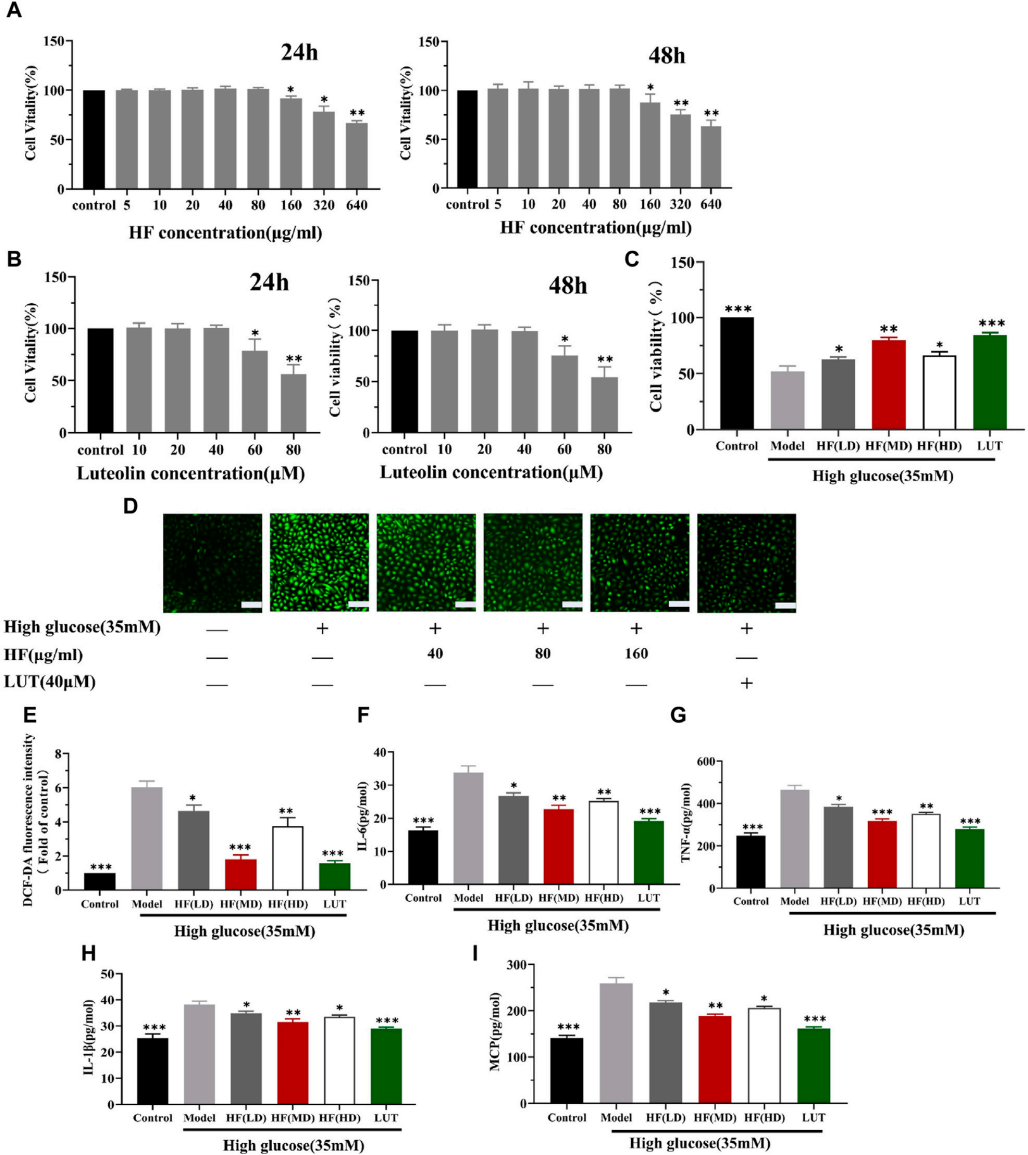
HF and luteolin attenuates high-glucose-induced ARPE-19 cell death and oxidative and inflammatory responses. **(A)** Comparison between different concentrations of HF and stimulation of ARPE-19 cells to media alone (control) for 24 h and 48 h. Cell viability is expressed as percentages of control. **(B)** Cells were incubated with luteolin in different doses (0, 10, 20, 40, 60, and 80 μM) for 24 h and 48 h **(C)** ARPE-19 cells cultured in high glucose (35 mM) for 48 h (model) or co-treated with HF (40, 80, and 160 μg/mL) (HF + high glucose), luteolin, and high glucose simultaneously for 24 h (luteolin + high glucose). **(D,E)** Detection of ARPE-19 cell ROS generation. Green fluorescence intensity represents ROS production. Scale bar, 20 µm for all images. **(F–I)** Measurement of inflammatory factors (IL-6, TNF-α, IL1-β, and MCP-1) by enzyme-linked immunosorbent assay. All data are presented as the mean ± SD. HF (LD): HF 40 μg/mL; HF (MD): HF 80 μg/mL; and HF (HD): HF 160 μg/mL *p < 0.05; **p < 0.01; and ***p < 0.001 *versus* model.

Under the concentration of HF < 160 μg/mL and luteolin <60 μM (Figures 9A, B), HF and luteolin have a non-toxic effect on ARPE-19 cells. When ARPE-19 cells were treated with HF or luteolin along with high-glucose treatment (35 mM), ARPE-19 cell viability was reserved. The result illustrated the protective effect of HF and luteolin on ARPE-19 cells in high-glucose conditions (Figure 9C).

#### 3.6.2 Reduction in ROS production

Since HF and luteolin were found to improve the reduction in ARPE-19 cell viability caused by high glucose, we investigated whether the beneficial effects were related to oxidative stress alleviation. Oxidative stress is activated due to ROS accumulation. Thus, DCFDA staining was performed to measure ROS production levels. Elevated levels of ROS were detected in cells cultured under high-glucose conditions. However, when ARPE-19 cells were treated with HF and luteolin, there was a reversal in ROS release (*p* all >0.05 vs. model) (Figure 9D). The result implied that HF and luteolin could attenuate retinal cells from oxidative stress damage.

#### 3.6.3 Amelioration inflammatory factor

Emerging evidence suggests that the connection between oxidative stress and inflammation is compact. Consequently, we calculated the secretion of IL-6, TNF-α, IL1-β, and MCP-1 in ARPE-19 cells. ELISA analysis revealed an increased inflammatory response with high-glucose stimulation, as evidenced by elevated levels of IL-6, TNF-α, IL1-β, and MCP-1. However, the generation of these factors was significantly reduced after HF and luteolin intervention (Figures 9E–H). The results suggested that HF and luteolin had a positive influence on ameliorating inflammation in high-glucose conditions.

#### 3.6.4 Decreased cell apoptosis

**FIGURE 10:**
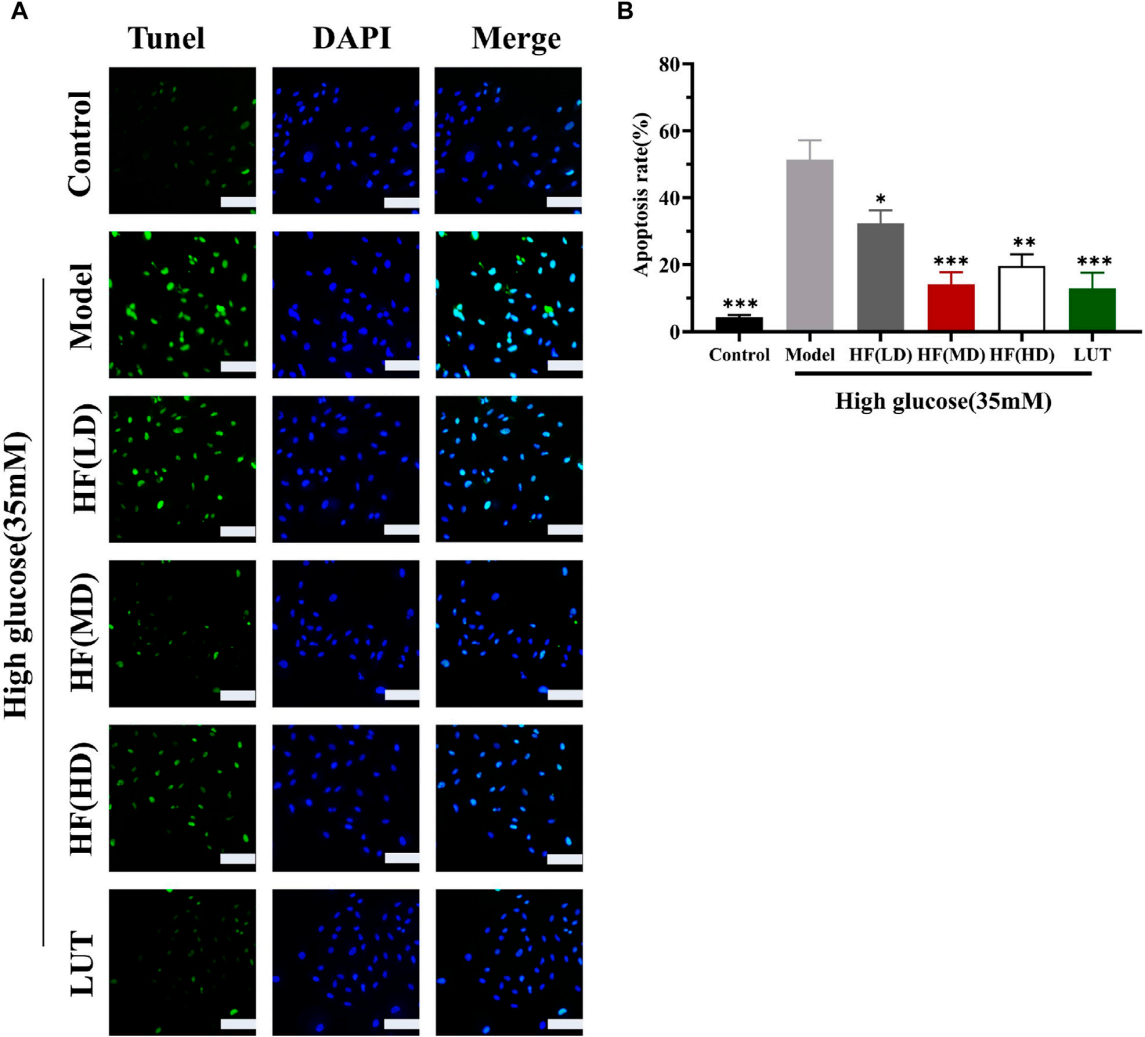
HF and luteolin relieved apoptosis in ARPE-19 cells **(A, B)**. The green color represents apoptotic cells, and the blue color symbolizes DAPI. All data are presented as the mean ± SD. HF (LD): HF 40 μg/mL; HF (MD): HF 80 μg/mL; and HF (HD): HF 160 μg/mL*p < 0.05; **p < 0.01; and ***p < 0.001 *versus* model. Scale bar, 20 µm for all images.

In WGCNA, ME-purple showed that apoptosis was strongly associated with DR. Figures 10A, B show that apoptosis was prominently increased in the model group. HF and luteolin treatment effectively ameliorated the occurrence rate of apoptosis in ARPE-19 cells. Our results revealed that HF and luteolin had effective roles in reducing inflammation in a high-glucose state.

### 3.7 Effect of HF and luteolin on SIRT1/P53 pathway activation: in vitro and *in vivo* assays

**FIGURE 11:**
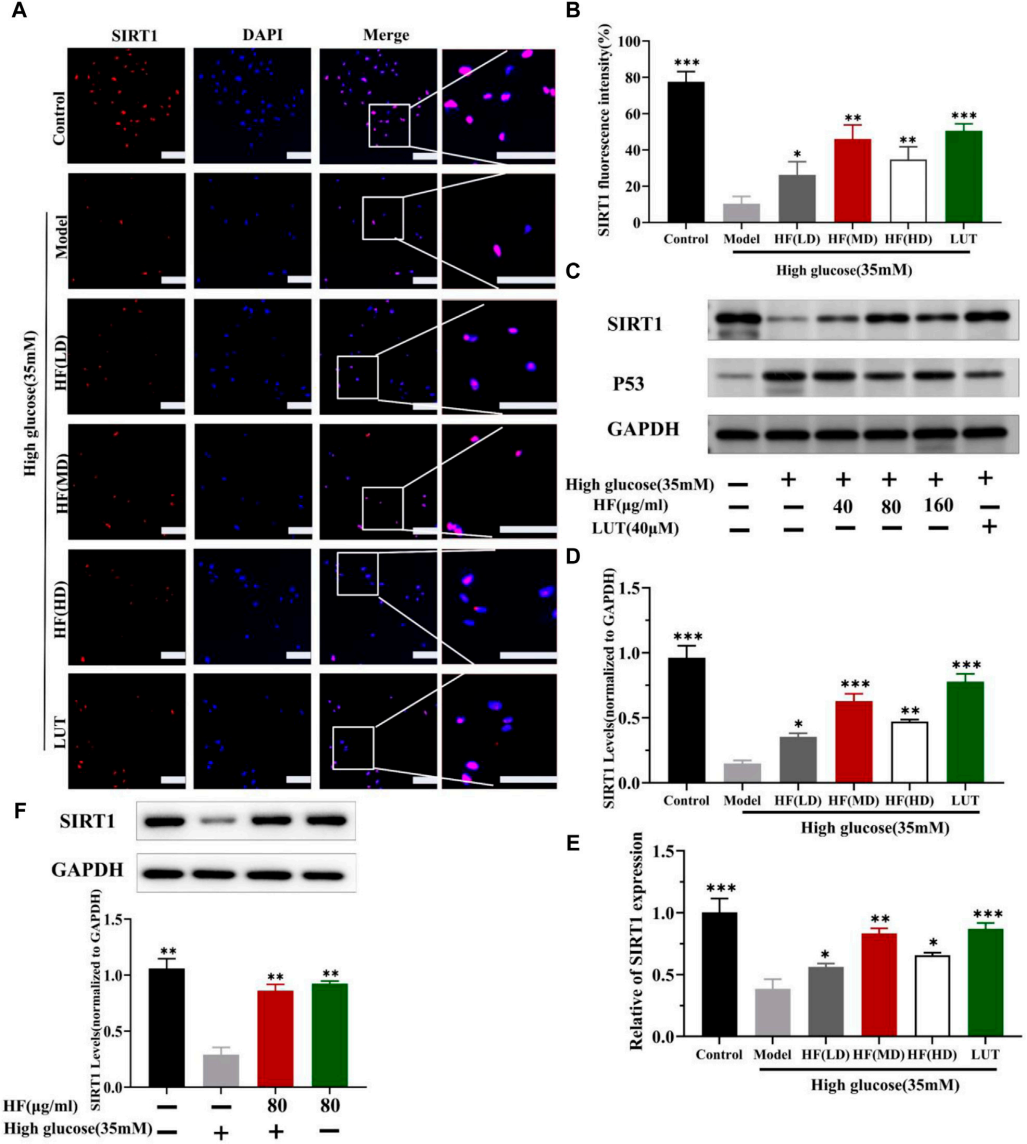
HF and luteolin promote the upregulation of SIRT1 in high-glucose-induced ARPE-19 cells. **(A)** Representative confocal microphotographs of ARPE-19 cells in different interventions groups, as described above. Cells were immunostained with SIRT1 (red) and DAPI (blue). Scale bar, 20 µm for all images. **(B)** Immunofluorescence expression analysis (n = 3). **(C)** Expression of SIRT1, P53, and GAPDH at protein levels in ARPE-19 cells. **(D, E)** Bar charts show the mean intensity of SIRT1 protein and mRNA quantified and normalized relative to GAPDH expression. **(F)** Expression of SIRT GAPDH at protein levels in ARPE-19 cells (5.5 mM and 35 mM). All data are presented as the mean ± SD. HF (LD): HF 40 μg/mL; HF (MD): HF 80 μg/mL; and HF (HD): HF 160 μg/mL*p < 0.05; **p < 0.01; and ***p < 0.001 *versus* model.

**FIGURE 12:**
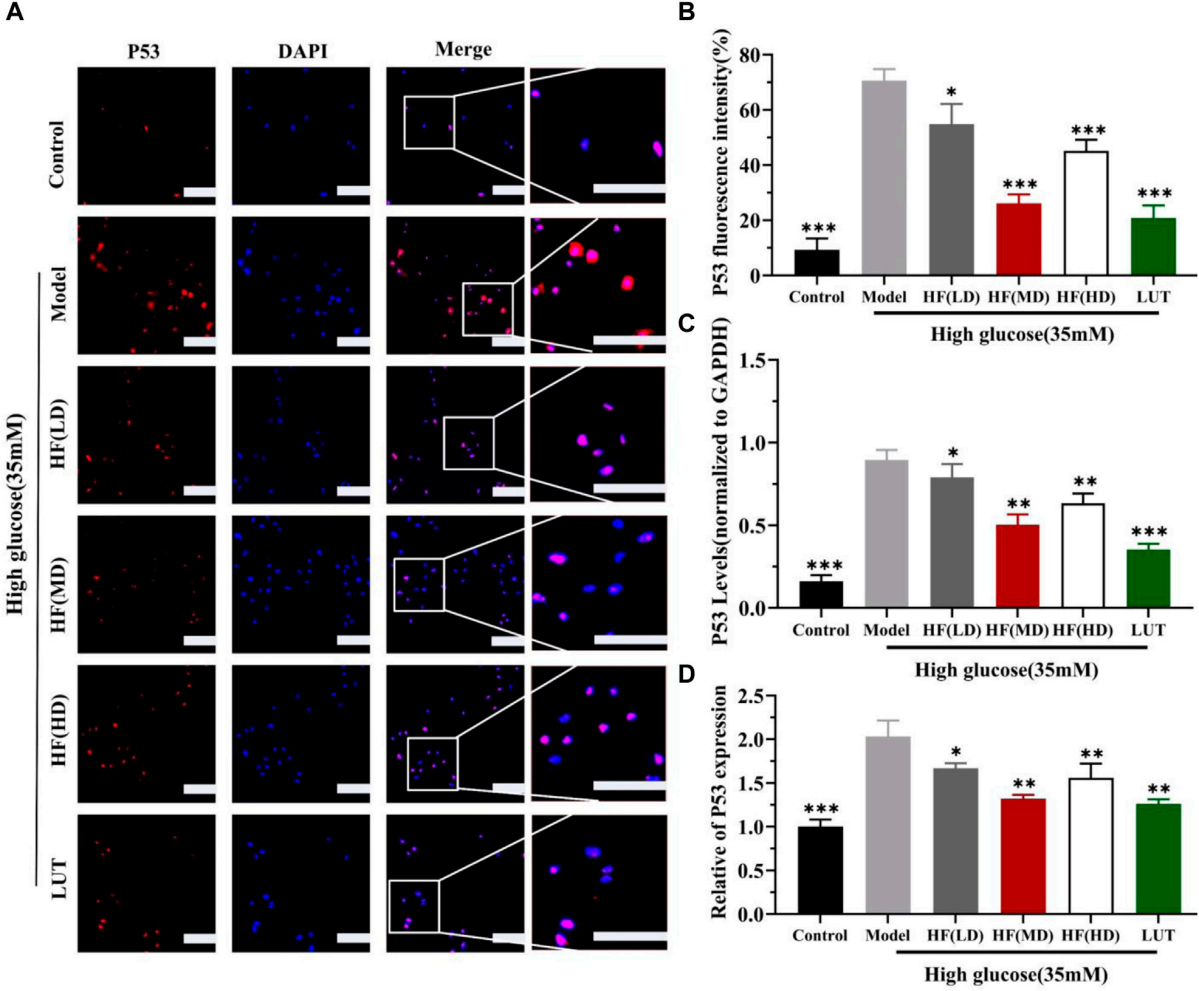
HF and luteolin lessen the expression of P53 *in vivo* experiment. **(A)** Representative confocal microphotographs of ARPE-19 cells in different interventions groups, as described above. Cells were immunostained with P53 (red) and DAPI (blue). Scale bar, 20 µm for all images. **(B)** Immunofluorescence expression analysis (n = 3). **(C, D)** Bar charts show the mean intensity of P53 protein and mRNA quantified and normalized relative to GAPDH expression. All data are presented as the mean ± SD. HF (LD): HF 40 μg/mL; HF(MD): HF 80 μg/mL; and HF (HD): HF 160 μg/mL*p < 0.05; **p < 0.01; and ***p < 0.001 *versus* model.

SIRT1 could reduce retinal damage and vasculopathy by inhibiting inflammatory responses and attenuating oxidative stress and apoptosis (Taurone et al., 2022). Immunofluorescence, Western blotting, and qRT-PCR (Figures 11A–E) results showed that SIRT1 expression was decreased in ARPE-19 cells induced by high glucose. However, HF and luteolin treatment increased SIRT1 expression, which suggested that upregulated SIRT1 contributed to DR improvement. P53 is also involved in DR development and progression (Ao et al., 2019). In this study (Figure 11C, Figures 12A–D), we found that under high-glucose conditions, P53 was significantly increased but decreased after intervention with HF and luteolin. The above results were consistent with an *in vivo* experiment (Figure 8C), which showed that increased SIRT1 and decreased P53 may be related to mechanisms underlying the amendatory influence of HF and luteolin against DR.

## 4 Discussion

In this study, we identified an effective anti-DR herbal formula with a novel mechanism. Based on the UPLC-Q-TOF-MS technology, 54 compounds were inferred from HF. Next, we screened 6 key active ingredients and 132 key targets. The finding revealed that DR treatment with HF is a multi-component and multi-targeted mode. In addition, we were surprised to find that medium HF showed the best pharmacodynamic effect in *in vitro* experiments. This observation can be attributed to two factors. First, the medium dose was determined as the optimal concentration based on the CCK-8 assay. Second, cells may have the ability to adapt to different dosages of stimuli. A low dose of HF may not be sufficient to trigger a cellular adaptive response, while a high dose may overstimulate the cells, leading to adverse effects. A moderate dose could stimulate the cells within an appropriate range, inducing an adaptive response that helps maintain cell viability.

In the WGCNA, we accidentally discovered two modules: ME-salmon and ME-purple, which represented negative regulation between “protein deacetylation” and “oxidative impairment.” Protein deacetylation is a post-translational DR denaturation process that involves the removal of acetyl groups from lysine residues in proteins (Jiang et al., 2023b). It is catalyzed by deacetylase enzymes, with the most renowned family being the sirtuins (SIRTs). Protein acetylation and deacetylation play crucial roles in regulating protein function, stability, and localization. In addition, they effectively influence various cellular processes, such as gene expression, DNA repair, metabolism, and stress response (Gan et al., 2023). The mechanism has been utilized to prevent cancer-related diseases, cardiovascular diseases, neurodegenerative conditions, and metabolic disorders (Naren et al., 2023). Oxidative impairment refers to a disequilibrium between ROS production and the cell’s ability to repair damage (Henrich et al., 2023). It can contribute to cellular dysfunction and tissue damage through multiple mechanisms, which include direct oxidative damage to biomolecules, inflammatory pathway activation, cellular signaling disruption, and mitochondrial function impairment (Martemucci et al., 2022). The current study shows that diabetes may result in genes’ epigenetic alterations for metabolic abnormalities (oxidative stress and inflammation) in the retina’s pathologic processes (Terao et al., 2022). DR formation may be related to altering global retinal acetylation histones and activating the acetylation–deacetylation machinery (Kowluru et al., 2021). Oxidative stress is a key factor in DR, but few studies monitor the modulation patterns between “protein deacetylation” and “oxidative stress.” Grasping the interaction between oxidative impairment and protein deacetylation is a promising research area. We should elucidate the specific targets and mechanisms involved in the process, providing insights into the development of therapeutic strategies to ameliorate oxidative stress-related diseases and promote retinal cell survival.

The hub genes identified in ME-salmon and ME-purple were SIRT1 and p53, respectively. SIRT1, a crucial NAD^+-dependent histone deacetylase, belongs to the SIRTs family. It is also known as silent information regulator 1 (Li et al., 2023) and plays a critical role in regulating cellular metabolism, stress response, DNA repair, and longevity. P53, often referred to as the “guardian of the genome,” is a noted tumor suppressor protein. P53 is proficient in maintaining genomic stability and preventing cancerous cell formation. The relationship between SIRT1 and p53 is complex and multifaceted. SIRT1 commands multiple cellular fates, which are predominantly linked to p53 activity (Ong and Ramasamy, 2018). SIRT1 can modulate cell senescence, cancer, and cellular reprogramming by deacetylating p53 (Brockmueller et al., 2023; Lo Cigno et al., 2023). The joint effect of SIRT1/P53 could inhibit ferroptosis and apoptosis (Zhang et al., 2023). In this study, we detected SIRT1 and P53 expression on immunofluorescence, Western blotting, and qRT-PCR aspects. The results revealed that upregulating SIRT1 and reducing P53 expression can play an efficient role in DR treatment.

Molecular docking is conducted to measure the binding mode and affinity between a ligand and its target molecule. The calculation results showed that luteolin displayed better binding activity with SIRT1 and P53. Luteolin is a natural flavonoid compound found in various plants and herbs and generates numerous health benefits and biological activities. Luteolin exhibits strong antioxidant and anti-inflammatory properties. In addition, luteolin could inhibit tumor cell growth, induce apoptosis, and improve endothelial function (Kang et al., 2019; Luo et al., 2019). Moreover, it possesses neuroprotective effects, suggesting its potential in treating neurodegenerative disorders such as Alzheimer’s and Parkinson’s diseases (Zhang Z. et al., 2022; He et al., 2023). Although luteolin was weakly correlated with DR in previous research, it manifests a positive impact on metabolic disorders like diabetes and obesity by modulating glucose metabolism and insulin sensitivity (Queiroz et al., 2021; Ge et al., 2022). In the ophthalmology field, luteolin could remedy corneal alkali burns and the epithelial–mesenchymal transition (EMT) of age-related macular degeneration (AMD). In addition, luteolin also delayed photoreceptor degeneration in a retinitis pigmentosa mouse model (Chen et al., 2022; Wang et al., 2023). In this study, we found that luteolin manifests a preferable effect on reducing blood sugar levels and improving weight loss, contrasting with HF. Furthermore, luteolin was creatively cultivated with high-glucose-induced ARPE-19 cells to simulate DR damage status *in vitro*. As a correlative effective demonstration, luteolin displayed non-toxicity to ARPE-19 cells at a certain concentration (<40 μM). It played an effective role in alleviating ROS accumulation, related inflammatory factors, and apoptotic cells induced by high glucose. In addition, these results expand our understanding of the mechanisms by which luteolin exerts beneficial effects on cellular health, providing further support for their potential application in the treatment of related diseases.

## 5 Conclusion

In this study, we focused on targeting protein acetylation and oxidative stress for the treatment of diabetic retinopathy. A Chinese herbal formula, He-Ying-Qing-Re formula (HF), and its bioactive compound, luteolin, could provide a promising treatment for diabetic retinopathy by suppressing protein acetylation and oxidative stress via the SIRT1/P53 pathway in retinal pigment epithelial cells. WGCNA-related measures and *in vitro*/*in vivo* approaches are used to validate our hypothesis. Additionally, our study offers novel insights into the mechanism of the SIRT1/P53 pathway in DR treatment in comparison with its traditional action. For this limitation, since our results mainly rely on *in vitro* and animal model experiments, more large-scale human clinical trials are recommended to validate these outcomes, as they offer insights into the efficacy and safety of HF and its bioactive compounds in human subjects. In conclusion, our study may lay a pre-clinical foundation for future anti-DR drug discovery.

## Data Availability

The original contributions presented in the study are included in the article/Supplementary Material; further inquiries can be directed to the corresponding authors.

## Abbreviations

ARPE-19, adult retinal pigment epithelial cell line-19; AUC, areas under the curve; DL, drug-like properties; DR, diabetic retinopathy; GO, gene ontology; HF, He-Ying-Qing-Re formula; IDF, International Diabetes Federation; OB, oral bioavailability; qRT-PCR, real-time quantitative reverse transcription PCR; ROS, reactive oxygen species; UPLC-Q-TOF-MS, ultra-performance liquid chromatography coupled with high-resolution mass spectrometry; WGCNA, weighted gene co-expression network analysis.
