# Supplementary material for: SIRT1/P53 in retinal pigment epithelial cells in diabetic retinopathy: a gene co-expression analysis and He-Ying-Qing-Re formula treatment
Source: Front Mol Biosci. 2024 Apr 3;11:1366020. doi: 10.3389/fmolb.2024.1366020 (PMC11021775; doi:10.3389/fmolb.2024.1366020)
Supplement: Supplementary file 1 [file DataSheet1.zip › Supplementary Materials/Supplementary Material 1.docx]

Supplementary Material 1

SIRT1/P53 in Retinal Pigment Epithelial Cells in diabetic retinopathy, a gene co-expression analysis and He-Ying-Qing-Re Formula Treatment

**Shuyan Zhang^*^, Jiajun Wu, Leilei Wang, Lin Mu, Xiaoyu Xu, Jiahui Li, Guoyi Tang, Guang Chen, Cheng Zhang, Yinjian Zhang, Yibin Feng**

*** Correspondence:**

Yinjian Zhang, e-mail: zhangyinj@126.com.

Yibin Feng, e-mail: yfeng@hku.hk.

# Supplementary Data

## Preparation of lyophilized powder of HF

Weigh 15 g of Flos Lonicerae, 9 g of Rhizoma Atractylodis macrocephalae, 12 g of Fructus Corni, 12 g of Fructus Lycii Barbari, 12 g of Cortex Moutan, 12 g of Semen Cuseutae, 12 g of Fructus Ligustri lucidi, 15 g of Radix Angelicae Sinensis, 15 g of Radix Scrophulariae, 9 g of Pericarpium Citri Reticulatae and 12 g of Radix Rehmanniae Exsiccata.Add 10 times the amount of water, soak for 30 mins, boil with a high fire, then decoct for 30 mins with a medium fire and then filter. Then, add 8 times the amount of water to the filtrate and decoct for 30 mins with a high fire. Filtering the filtration, combine the two filtrates, concentrate to a small volume, lyophilize, and obtain. (Total 135 g of herbs, 57.9 g of lyophilized powder)

Take about 2 g of lyophilized powder sample, weigh it precisely. Then put it in a 50ml stoppered conical flask, add 10ml of methanol precisely with ultrasonic treatment (power 250 W, frequency 40 kHz) for 30 minutes in the appropriate amount. Let it cool down and weigh it. Take 2ml and centrifuge (12000 rpm) for 5 minutes, take the supernatant.

## Chromatographic conditions of HF

Chromatographic column: Waters ACQUITY UPLC BEH C18 (2.1×100 mm, 1.7 µm); column temperature: 30℃; flow rate: 0.3ml/min; injection volume: 2 µl; detection wavelength: 260 nm; mobile phase ratio: acetonitrile in A-phase, 0.1% formic acid aqueous solution in B-phase, and the gradient is shown in Table 1.

## Setting mass spectrometry conditions

Mass spectrometry detection mode: ESI-Negative/ Positive ion mode; Mass spectrometry parameters is shown in Table 2.

## Preparation of control solution

Take the appropriate amount of Luteolin control product, weigh it precisely, add methanol to make a solution containing 0.8 μg per 1ml.

## Chromatographic conditions of control product

Column: Agilent ZORBAX RRHD Extend-C18 (3.0×100 mm, 1.8 µm); Column temperature:30℃; Flow rate:0.3 ml/min; Injection volume:2 µl; Detection wavelength: 190-400nm; Mobile phase: Acetonitrile in A-phase, 0.1% formic acid aqueous solution in B-phase, the gradient is shown in Table 3.

## Control mass spectrometry conditions

Detection mode: ESI-Positive/Negative ion mode; ESI source parameters: Ion source is electrospray ion (ESI), Ion Source Temperature (Temperature TEM) 550°C, Spray Gas (Ion Source Gas1 GS1) 50.0 psi, Auxiliary Heating Gas (Ion Source Gas2) 40.0 psi, Curtain Gas CUR 20.0 psi, Ionization Voltage (IonSpray Voltage IS) -20.0 psi. GS2) 40.0 psi, Curtain Gas CUR (CUR) 20.0 psi, and IonSpray Voltage IS (IonSpray Voltage IS) -4500 V. The MRM parameters of each compound are shown in Table 4.

# Supplementary Tables

**Table 1** Mobile phase gradients

| Time (min) | A% | B% |
| --- | --- | --- |
| 0～5 | 3 | 97 |
| 5～8 | 3～8 | 97～92 |
| 8～15 | 8 | 92 |
| 15～35 | 8～25 | 92～75 |
| 35～45 | 25～45 | 75～55 |
| 45～50 | 45～95 | 55～5 |
| 50~53 | 95 | 5 |
| 53~53.1 | 95~3 | 5~97 |
| 53.1～56 | 3 | 97 |

**Table 2** Mass parameters (Sciex Triple TOF 4600 LC-MS)

| MS parameters | reference point | MS/MS parameters | reference point |
| --- | --- | --- | --- |
| TOF mass range | 50～1700 | MS/MS mass range | 50～1250 |
| Ion Source Gas 1（psi） | 50 | Declustering Potential（V） | 100 |
| Ion Source Gas 2（psi） | 50 | Collision Energy（eV） | ±40 |
| Curtain Gas（psi） | 35 | Collision Energy Spread（eV） | 20 |
| Ion Spray Voltage Floating (V) | -4500/5000 | Ion Release Delay（ms） | 30 |
| Ion Source Temperature (°C) | 500 | Ion Release Width（ms） | 15 |
| Declustering Potential（V） | 100 |  |  |
| Collision Energy（eV） | 10 |  |  |

**Table 3 Mobile phase gradients**

| Time (min) | A% | B% |
| --- | --- | --- |
| 0～3 | 40 | 60 |
| 3～9 | 40～60 | 60～40 |
| 9～15 | 60 | 40 |

**Table 4** MRM parameters

| Compound | Precursor Ion（*m*/*z*） | Product Ion（*m*/*z*） | DP（V） | CE（V） | EP（V） | CXP（V） |
| --- | --- | --- | --- | --- | --- | --- |
| Luteolin | 285.0 | 133.0^*^ | -100  -95 | -34 | -9 | -8 |
|  |  | 151.3 |  | -36 | -9 | -3 |

Note: "*" quantifies ion pairs.
