# Supplementary material for: SIRT1/P53 in retinal pigment epithelial cells in diabetic retinopathy: a gene co-expression analysis and He-Ying-Qing-Re formula treatment
Source: Front Mol Biosci. 2024 Apr 3;11:1366020. doi: 10.3389/fmolb.2024.1366020 (PMC11021775; doi:10.3389/fmolb.2024.1366020)
Supplement: Supplementary file 1 [file DataSheet1.zip › Supplementary Materials/Supplementary Material 3.docx]

Supplementary Material 3

SIRT1/P53 in Retinal Pigment Epithelial Cells in diabetic retinopathy, a gene co-expression analysis and He-Ying-Qing-Re Formula Treatment

**Shuyan Zhang^*^, Jiajun Wu, Leilei Wang, Lin Mu, Xiaoyu Xu, Jiahui Li, Guoyi Tang, Guang Chen, Cheng Zhang, Yinjian Zhang, Yibin Feng**

*** Correspondence:**

Yinjian Zhang, e-mail: zhangyinj@126.com.

Yibin Feng, e-mail: [yfeng@hku.hk](mailto:yfeng@hku.hk).

# Supplementary Data

##########################################################

Setwd ('C:/Users/SCM/Desktop/Run data')

# The following setting is important, do not omit.

Options (stringsAsFactors = FALSE);

expro=read. Csv ('counts_anno.xls', sep = '\t', row.names = 1)

dim(expro)

##########################################################

m.vars=apply (expro,1, var)

expro.upper=expro[which(m.vars>quantile(m.vars, probs = seq(0, 1,0.2))[4]),]

dim(expro.upper)

write. Table (expro. upper, file="geneInput_variancetop0.25.txt", sep='\t',quote=F,row.names=T)

datExpr0 = as.data.frame(t(expro.upper));

####################################################################

Library (WGCNA)

####################################################################

gsg = goodSamplesGenes(datExpr0, verbose = 3);

gsg$allOK

####################################################################

sampleTree = hclust(dist(datExpr0), method = "average");

# Plot the sample tree: Open a graphic output window of size 12 by 9 inches

# The user should change the dimensions if the window is too large or too small.

sizeGrWindow(12,9)

#pdf (file = "Plots/sampleClustering.pdf", width = 12, height = 9);

Par (cex = 0.45);

Par (mar = c (0,4,2,0))

Plot (sampleTree, main = "Sample clustering to detect outliers",

sub="", xlab="",

cex.lab = 1.5, cex.axis = 1.5, cex.main = 2)

# Plot a line to show the cut

abline(h = 275000, col = "red");

# Determine cluster under the line

clust = cutreeStatic(sampleTree, cutHeight = 275000, minSize = 2)

table(clust)

# clust 1 contains the samples we want to keep.

keepSamples = (clust==1)

datExpr = datExpr0[keepSamples,]

nGenes = ncol(datExpr)

nSamples = nrow(datExpr)

# The following setting is important, do not omit.

Options (stringsAsFactors = FALSE);

# See note above.

enableWGCNAThreads()

powers = c (c (1:10), seq (from = 12, to=20, by=2))

# Call the network topology analysis function

sft = pickSoftThreshold(datExpr, powerVector = powers, verbose = 5)

####################################################################

# Plot the results:

sizeGrWindow (9, 5)

par (mfrow = c (1,2));

cex1 = 0.9;

# Scale-free topology fit index as a function of the softthresholding

power

plot(sft$fitIndices[,1], -sign(sft$fitIndices[,3])*sft$fitIndices[,2],

xlab="Soft Threshold (power)", ylab="Scale Free Topology Model

Fit,signed R^2",type="n",

main = paste ("Scale independence"));

text(sft$fitIndices[,1], -sign(sft$fitIndices[,3])*sft$fitIndices[,2],

labels=powers,cex=cex1,col="red");

# this line corresponds to using an R^2 cut-off of h

abline (h=0.8, col="red")

####################################################################

# Mean connectivity as a function of the soft-thresholding power

plot(sft$fitIndices[,1], sft$fitIndices[,5],

xlab="Soft Threshold (power)", ylab="Mean Connectivity",

type="n",

main = paste ("Mean connectivity"))

text(sft$fitIndices[,1], sft$fitIndices[,5], labels=powers,

cex=cex1, col="red")

# here we define the adjacency matrix using soft thresholding with

beta=14

ADJ1=abs(cor(datExpr,use="p"))^14

# When you have relatively few genes (<5000) use the following code

k=as.vector(apply(ADJ1,2,sum, na.rm=T))#⼆二

# Plot a histogram of k and a scale free topology plot

sizeGrWindow (10,5)

par (mfrow=c (1,2))

hist(k)

scaleFreePlot(k, main="Check scale free topology\n")

#=========================================================

softPower = 14;

adjacency = adjacency (datExpr, power = softPower)

TOM = TOMsimilarity(adjacency);

dissTOM = 1-TOM

#####################################################################

geneTree = hclust(as.dist(dissTOM), method = "average");

# Plot the resulting clustering tree (dendrogram)

sizeGrWindow(12,12)

plot (geneTree, xlab="", sub="", main = "Gene clustering on TOM-based

dissimilarity",

labels = FALSE, hang = 0.04);

#####################################################################

minModuleSize =340;

# Module identification using dynamic tree cut:

dynamicMods = cutreeDynamic(dendro = geneTree, distM = dissTOM,

deepSplit = 2, pamRespectsDendro =

FALSE,

minClusterSize = minModuleSize);

table(dynamicMods)

dynamicColors = labels2colors(dynamicMods)

table(dynamicColors)

#####################################################################

sizeGrWindow(8,12)

plotDendroAndColors(geneTree, dynamicColors, "Dynamic Tree Cut",

dendroLabels = FALSE, hang = 0.03,

addGuide = TRUE, guideHang = 0.05,

main = "Gene dendrogram and module colors")

#===================================================================

# calculate eigengenes

MEList = moduleEigengenes(datExpr, colors = dynamicColors)

MEs = MEList$eigengenes# Calculate dissimilarity of module eigengenes

MEDiss = 1-cor (MEs); # Cluster module eigengenes

METree = hclust(as.dist(MEDiss), method = "average");

# Plot the result

sizeGrWindow(7, 6)

plot (METree, main = "Clustering of module eigengenes",

xlab = "", sub = "")

MEDissThres = 0.2

###################################################################

abline(h=MEDissThres, col = "red")

merge = mergeCloseModules (datExpr, dynamicColors, cutHeight =

MEDissThres, verbose = 3)

# The merged module colors

mergedColors = merge$colors;

# Eigengenes of the new merged modules:

mergedMEs = merge$newMEs;

#===================================================================

sizeGrWindow(12, 9)

#pdf (file = "Plots/geneDendro-3.pdf", wi = 9, he = 6)

plotDendroAndColors(geneTree, cbind(dynamicColors, mergedColors),

c ("Dynamic Tree Cut", "Merged dynamic"),

dendroLabels = FALSE, hang = 0.03,

addGuide = TRUE, guideHang = 0.05)

#dev.off ()

# Rename to moduleColors

moduleColors = mergedColors

#===================================================================

# Define numbers of genes and samples

nGenes = ncol(datExpr);

nSamples = nrow(datExpr);

#######################################################

MEs0 = moduleEigengenes(datExpr,moduleColors)$eigengenes

MEs = orderMEs(MEs0)

######################################################

# Save the result of values of module Eigengenes

MEList2= moduleEigengenes(datExpr, colors =moduleColors)[[1]]

MEs2<- MEList$eigengenes

colors2 <-as.character(moduleColors)

datKME<-signedKME(datExpr, MEList2)

geneInfo0 <-data.frame (geneSymbol=rownames(t(datExpr)),moduleColors=mergedColors, datKME)

write.table(geneInfo0, "Gene-information1.xls", sep="\t", row.names=F, quote=F)

###########################################################################

#=========================================================#

# Pearson’s R square and P value between modules

#=========================================================#

PearsonsR <- signif(cor(MEList2,use="p"))

RSquare <- PearsonsR^2

nSamples=nrow(PearsonsR)

PearsonsP <-corPvalueStudent(PearsonsR,nSamples)

############################################################

install.packages("scatterplot3d")

library(scatterplot3d)

##################################################################

#=========================================================

# Geometric data shown in 3D scattering

cmd1=cmdscale(as.dist(dissTOM),3)

pairs (cmd1, col=as.character(moduleColors), main="MDS plot",pch=16)

par (mfrow=c (1,1), mar=c (4,3,2,3) +0.1, cex=0.85, pch=16, lwd=3)

s3d <- scatterplot3d (cmd1, color=moduleColors,angle=210,xlab="Scaling Axis 1", ylab="Scaling Axis 2", zlab="Scaling Axis 3",type = "p")

my.lm <- lm(cmd1[,3] ~ cmd1[,1] + cmd1[,2])

s3d$plane3d(my.lm,col="blue4")

################################################################################

#=========================================================

# Pairwise illustration of gene module correlation

#=========================================================

pairs(datKME[1:500,],

panel= function(x,y){points(x,y,col = c("black", "blue","green","greenyellow","grey","magenta","pink","purple","salmon","tan")

,pch=16,cex=1.4)

abline(lm(y~x), col='black', lwd=2.5)

text (0.5,0.8, labels = paste ('R2=', round((cor(x,y))^2,2)),

col='red', cex=1)})

#=========================================================#

#==========================================================

########################## ####### #########################

# Select module

module = "purple";

# Select module probes

probes = names(datExpr)

inModule = (moduleColors==module);

modProbes = probes[inModule];

IMConn = softConnectivity(datExpr[, modProbes],power=6);

dat1=datExpr[inModule]

datExp_IMConn <-data.frame(IMConn,t(dat1))

datExp_IMConn=data.frame(datExp_IMConn)

write.table(datExp_IMConn,

file = paste ("Intramodule_connectivity-purple",module," .txt"),sep='\t')

######################################################

nTop = 70

top = (rank(-IMConn) <= nTop)

dat2=t(datExp_IMConn)

dat2<-data.frame(dat2)

dat3<-dat2[top]

dat3<-t(dat3)

dat3<-data.frame(dat3)

write.table(dat3, file =paste("Intramodule_connectivity purple",module,"-top70.txt"),sep='\t')

==========================================================

########################## ####### #########################

# Select module

module = "salmon";

# Select module probes

probes = names(datExpr)

inModule = (moduleColors==module);

modProbes = probes[inModule];

IMConn = softConnectivity(datExpr[, modProbes],power=6);

dat1=datExpr[inModule]

datExp_IMConn <-data.frame(IMConn,t(dat1))

datExp_IMConn=data.frame(datExp_IMConn)

write.table(datExp_IMConn,

file = paste ("Intramodule_connectivity-samlon", module," .txt"), sep='\t')

###############################################################

nTop = 70

top = (rank(-IMConn) <= nTop) dat2=t(datExp_IMConn)

dat2<-data.frame(dat2)

dat3<-dat2[top]

dat3<-t(dat3)

dat3<-data.frame(dat3)

write.table(dat3, file =paste("Intramodule_connectivity salmon dule,"-top70.txt"),sep='\t')
