# Supplementary material for: SIRT1/P53 in retinal pigment epithelial cells in diabetic retinopathy: a gene co-expression analysis and He-Ying-Qing-Re formula treatment
Source: Front Mol Biosci. 2024 Apr 3;11:1366020. doi: 10.3389/fmolb.2024.1366020 (PMC11021775; doi:10.3389/fmolb.2024.1366020)
Supplement: Supplementary file 1 [file DataSheet1.zip › Supplementary Materials/Supplementary Material 4.docx]

Supplementary Material 4

SIRT1/P53 in Retinal Pigment Epithelial Cells in diabetic retinopathy, a gene co-expression analysis and He-Ying-Qing-Re Formula Treatment

**Shuyan Zhang^*^, Jiajun Wu, Leilei Wang, Lin Mu, Xiaoyu Xu, Jiahui Li, Guoyi Tang, Guang Chen, Cheng Zhang, Yinjian Zhang, Yibin Feng**

*** Correspondence:**

Yinjian Zhang, e-mail: zhangyinj@126.com.

Yibin Feng, e-mail: [yfeng@hku.hk](mailto:yfeng@hku.hk).

# Supplementary Data

**Table 1** Identification of Chemical Components in HF

| Number | Time  (min) | adatoms |  | m/z actual value | m/z theoretica1  value | ppm | molecular formulae | molecular mass | chemical compound | MS/MS | peak area | Database comparison | belongingness |
| --- | --- | --- | --- | --- | --- | --- | --- | --- | --- | --- | --- | --- | --- |
| 1 | 1.14 | [M-H]^-^ |  | 191.0200 | 191.0197 | 1.4 | C_6_H_8_O_7_ | 192.03 | Citric acid | 191.0195;129.0189;111.0085;87.0087 | 8270787 | *√* | S |
| 2 | 1.3 | [M+FA-H]^-^ |  | 407.1203 | 407.1195 | 2 | C_15_H_22_O_10_ | 362.12 | Catalpol | 407.1176;361.1136;199.0607;169.0501;151.0395 | 3621193 | *√* | S/X |
| 3 | 1.81 | [M-H]^-^ |  | 169.0147 | 169.0142 | 2.7 | C_7_H_6_O_5_ | 170.02 | Gallic acid | 169.0149;125.0255;79.0192 | 2981312 | *√* | D |
| 4 | 2.28 | [M+FA-H]^-^ |  | 391.1257 | 391.1246 | 2.8 | C_15_H_22_O_9_ | 346.13 | Aucubin | 391.1249;345.1187;183.0657;165.0553;139.0396 | 1469695 | *√* | S/X |
| 5 | 2.91 | [M+H]^+^ |  | 127.0382 | 127.039 | -6.1 | C_6_H_6_O_3_ | 126.03 | 5-Hydroxymethyl-2-Furaldehyde | 109.0268;81.0316 | 233205 | *√* | Z |
| 6 | 2.99 | [M+FA-H]^-^ |  | 731.2277 | 731.2251 | 3.5 | C_27_H_42_O_20_ | 686.23 | Rehmannioside D | 685.2226;505.1569;341.1068;263.0756;221.0654;179.0542 | 1002296 | *√* | S |
| 7 | 4.57 | [M+FA-H]^-^ |  | 409.1364 | 409.1352 | 3.1 | C_15_H_24_O_10_ | 364.14 | Harpagide | 363.1286;201.0759;183.0662;139.0399 | 3506620 | *√* | X |
| 8 | 5.29 | [M+FA-H]^-^ |  | 393.1403 | 393.1402 | 0.2 | C_15_H_24_O_9_ | 348.14 | Ajugol | 393.1427;347.1366;167.0715;149.0609;127.0401 | 2106066 | *√* | S |
| 9 | 6.4 | [M-H]^-^ |  | 353.0897 | 353.0878 | 5.4 | C_16_H_18_O_9_ | 354.10 | Neochlorogenic acid | 353.0903;191.0570;179.0362;135.0456 | 1109396 |  | J/T/G/Q/B |
| 10 | 6.76 | [M+H]^+^ |  | 205.0959 | 205.0972 | -6.1 | C_11_H_12_N_2_O_2_ | 204.09 | L-tryptophan | 188.0661;146.0580;118.0614 | 147801 | *√* | G |
| 11 | 7.36 | [M-H]^-^ |  | 515.1429 | 515.1406 | 4.4 | C_22_H_28_O_14_ | 516.15 | 4-O-(3'-O-caffeoyl glucosyl) quinic acid | 515.1423;353.0885;191.0560;179.0345 | 1664468 |  | T |
| 12 | 8.84 | [M-H]^-^ |  | 299.1151 | 299.1136 | 4.9 | C_14_H_20_O_7_ | 300.12 | Salidroside | 119.0499;89.0234;71.0145 | 592049 | *√* | N |
| 13 | 9.2 | [M-H]^-^ |  | 433.0996 | 433.0988 | 1.9 | C_17_H_22_O_13_ | 434.11 | Ligulucidumoside C-C2H4 | 433.0980;209.0459;177.0194;165.0557 | 3936395 |  | N |
| 14 | 10.54 | [M-H]^-^ |  | 353.0877 | 353.0878 | -0.3 | C_16_H_18_O_9_ | 354.10 | Chlorogenic acid | 353.0855;191.0554;179.0338;161.0232;127.0389 | 9154616 | *√* | J/T/G/Q/B |
| 15 | 10.72 | [M+FA-H]^-^ |  | 451.1459 | 451.1457 | 0.4 | C_17_H_26_O_11_ | 406.15 | Morroniside | 405.1418;353.0890;243.0876;191.0555;155.0356 | 7045149 | *√* | Z |
| 16 | 10.9 | [M-H]^-^ |  | 495.1514 | 495.1508 | 1.2 | C_23_H_28_O_12_ | 496.16 | Oxypaeoniflora | 495.1500;465.1369;333.0973;165.0561;137.0242 | 1894493 | *√* | D |
| 17 | 11.58 | [M-H]^-^ |  | 353.0878 | 353.0878 | 0 | C_16_H_18_O_9_ | 354.10 | Cryptochlorogenic acid | 353.0935;191.0567;179.0359;173.0465;135.0455 | 2191862 | *√* | J/T/G/Q/B |
| 18 | 11.69 | [M+H]^+^ |  | 796.347 | 796.3499 | -3.6 | C_37_H_53_N_3_O_16_ | 795.34 | Lycibarbarspermidine E | 796.3474;634.2979;472.2747;382.1438;220.0936 | 313573 |  | Q |
| 19 | 12.14 | [M-H]^-^ |  | 389.1084 | 389.1089 | -1.4 | C_16_H_22_O_11_ | 390.12 | Secoxyloganic acid | 389.1100;345.1182;209.0460;183.0667;65.0555 | 8408189 | *√* | J |
| 20 | 12.57 | [M-H]^-^ |  | 373.1145 | 373.114 | 1.3 | C_16_H_22_O_10_ | 374.12 | Secologanic acid | 373.1154;193.0512;149.0603;119.0343;97.0293 | 13595394 | *√* | J |
| 21 | 15.69 | [M+FA-H]^-^ |  | 403.1251 | 403.1246 | 1.3 | C_16_H_22_O_9_ | 358.13 | Sweroside | 403.1241;357.1199;195.0666;179.0560;125.0258 | 4436474 | *√* | J/Z |
| 22 | 16.99 | [M+FA-H]^-^ |  | 435.1518 | 435.1508 | 2.3 | C_17_H_26_O_10_ | 390.15 | Loganin | 435.1523;227.0916;127.0396;101.0241 | 10856707 | *√* | J/Z |
| 23 | 18.78 | [M+FA-H]^-^ |  | 525.1647 | 525.1614 | 6.4 | C_23_H_28_O_11_ | 480.16 | Paeoniflorin | 479.1625;449.1477;327.1088;165.0556;121.0306 | 8332078 | *√* | D |
| 24 | 21.06 | [M-H]^-^ |  | 403.1263 | 403.1246 | 4.3 | C_17_H_24_O_11_ | 404.13 | Secoxyloganin | 403.1257;371.1027;223.0637;165.0561;121.0295 | 5080972 | *√* | J |
| 25 | 21.24 | [M+FA-H]^-^ |  | 433.1375 | 433.1352 | 5.4 | C_17_H_24_O_10_ | 388.14 | Vogeloside | 433.1335;387.1267;225.0755;179.0555;155.0349 | 10934059 |  | J |
| 26 | 23.92 | [M-H]^-^ |  | 595.1324 | 595.1305 | 3.3 | C_26_H_28_O_16_ | 596.14 | Quercetin-3-O-β-D-apifuranosyl-(1→2)-β-D-galactoside | 595.1317;300.0271;271.0240;255.0293 | 1543698 |  | T |
| 27 | 23.98 | [M+H]^+^ |  | 538.2261 | 538.2283 | -4.1 | C_26_H_35_NO_11_ | 537.22 | L-Phenylalaninosecologanin | 538.2305;376.1768;358.1675;298.1443;228.0989;211.0959 | 1176161 |  | J |
| 28 | 24.63 | [M-H]^-^ |  | 463.0900 | 463.0882 | 3.9 | C_21_H_20_O_12_ | 464.10 | Hyperoside | 463.0931;300.0292;271.0251;255.0302 | 3206564 | *√* | T |
| 29 | 24.91 | [M-H]^-^ |  | 609.1501 | 609.1461 | 6.6 | C_27_H_30_O_16_ | 610.15 | Rutin | 609.1479;300.0272;271.0250;255.0297;178.9987 | 1511392 | *√* | T/Z |
| 30 | 25.26 | [M-H]^-^ |  | 463.0875 | 463.0882 | -1.5 | C_21_H_20_O_12_ | 464.10 | Isoquercitrin | 463.0887;300.0274;271.0242;255.0293 | 1509690 | *√* | T |
| 31 | 25.58 | [M-H]^-^ |  | 569.1532 | 569.1512 | 3.5 | C_25_H_30_O_15_ | 570.16 | Oleuropeinic acid | 569.1503;389.0845;363.1074;331.0812;151.0399 | 711150 |  | N |
| 32 | 25.69 | [M-H]^-^ |  | 447.0956 | 447.0933 | 5.2 | C_21_H_20_O_11_ | 448.10 | Luteoloside | 447.0951;327.0517;285.0409;256.0370 | 332995 | *√* | J |
| 33 | 27.15 | [M-H]^-^ |  | 623.2015 | 623.1981 | 5.4 | C_29_H_36_O_15_ | 624.21 | Verbascoside | 623.1997;461.1673;179.0348;161.0241 | 2151393 | *√* | S |
| 34 | 27.61 | [M-H]^-^ |  | 579.1746 | 579.1719 | 4.6 | C_27_H_32_O_14_ | 580.18 | Naringin | 579.1776;313.0731;295.0617;271.0622;177.0189;151.0041 | 5326329 | *√* | C |
| 35 | 27.73 | [M-H]^-^ |  | 515.1219 | 515.1195 | 4.7 | C_25_H_24_O_12_ | 516.13 | Isochlorogenic acid B | 515.124;353.0897;191.0564;179.0352;173.0460 | 329132 | *√* | J/T/Q/B |
| 36 | 28.03 | [M-H]^-^ |  | 515.1219 | 515.1195 | 4.7 | C_25_H_24_O_12_ | 516.13 | Isochlorogenic acid A | 515.1201;353.0892;191.0557;179.0345;135.0442 | 876889 | *√* | J/T/Q/B |
| 37 | 28.38 | [M-H]^-^ |  | 623.2008 | 623.1981 | 4.3 | C_29_H_36_O_15_ | 624.21 | Isoacteoside | 623.2040;461.1691;179.0361;161.0255 | 800498 | *√* | S |
| 38 | 28.65 | [M-H]^-^ |  | 685.2376 | 685.2349 | 3.9 | C_31_H_42_O_17_ | 686.24 | Specnuezhenide | 685.2364;523.1839;453.1415;299.1138;223.0597 | 5110751 | *√* | N |
| 39 | 29.55 | [M-H]^-^ |  | 541.1592 | 541.1563 | 5.4 | C_24_H_30_O_14_ | 542.16 | Cornuside | 541.1609;347.0807;277.0671;169.0150;125.0238 | 1925460 | *√* | Z |
| 40 | 29.56 | [M-H]^-^ |  | 609.1872 | 609.1825 | 7.7 | C_28_H_34_O_15_ | 610.19 | Hesperidin | 609.1832;343.0800;325.0707;301.0718;286.0483 | 11053400 | *√* | C |
| 41 | 30.06 | [M-H]^-^ |  | 515.1214 | 515.1195 | 3.7 | C_25_H_24_O_12_ | 516.13 | Isochlorogenic acid C | 515.1311;353.0924;191.0568;179.0361;173.0466 | 811561 | *√* | J/T/Q/B |
| 42 | 31.55 | [M-H]^-^ |  | 783.2766 | 783.2717 | 6.3 | C_36_H_48_O_19_ | 784.28 | Angoroside C | 783.2791;607.2308;589.2212;193.0500;175.0393 | 2278007 | *√* | X |
| 43 | 33.93 | [M-H]^-^ |  | 285.0412 | 285.0405 | 2.6 | C_15_H_10_O_6_ | 286.05 | Luteolin | 285.0405;175.0403;151.0027;133.0303;107.0163 | 65795 | *√* | J |
| 44 | 35.08 | [M+H]^+^ |  | 167.0695 | 167.0703 | -4.6 | C_9_H_10_O_3_ | 166.06 | Paeonol | 167.0708;149.0576;121.0637;91.0509;77.0360 | 3483822 | *√* | D |
| 45 | 36.52 | [M+FA-H]^-^ |  | 539.1818 | 539.177 | 8.9 | C_24_H_30_O_11_ | 494.18 | Harpagoside | 493.1748;345.1179;165.0550;147.0451;103.0551 | 3708195 | *√* | X |
| 46 | 38.58 | [M+FA-H]^-^ |  | 629.192 | 629.1935 | 7 | C_30_H_32_O_12_ | 584.19 | Benzoylpaeoniflorin | 583.1863;553.1771;431.1350;165.0566;121.0298 | 5285840 | *√* | D |
| 47 | 39.5 | [M-H]^-^ |  | 723.2188 | 723.2142 | 6.4 | C_33_H_40_O_18_ | 724.22 | Melitidin | 723.2183;417.1191;402.0929;387.0711;359.0774 | 618228 |  | C |
| 48 | 43.91 | [M+H]^+^ |  | 403.1363 | 403.1387 | -6.1 | C_21_H_22_O_8_ | 402.13 | Nobiletin | 403.1369;388.1137;373.0886;355.0817 | 1468594 | *√* | C |
| 49 | 44.97 | [M+H]^+^ |  | 433.1483 | 433.1493 | -2.3 | C_22_H_24_O_9_ | 432.14 | 3',4',3,5,6,7,8-Heptamethoxyflavone | 433.1504;418.1266;403.1006;385.0884;373.0501 | 3003821 |  | C |
| 50 | 45.54 | [M+H]^+^ |  | 249.1468 | 249.1485 | -6.9 | C_15_H_20_O_3_ | 248.14 | Atractylenolide Ⅲ | 231.1362;175.0741;163.0748;105.0671 | 52197 | *√* | B |
| 51 | 45.85 | [M+H]^+^ |  | 373.1265 | 373.1282 | -4.5 | C_20_H_20_O_7_ | 372.12 | Tangeretin | 373.1303;358.1001;343.0789;325.0619;297.0697 | 324291 | *√* | C |
| 52 | 46.8 | [M+H]^+^ |  | 191.1062 | 191.1067 | -2.4 | C_12_H_14_O_2_ | 190.10 | 3-n-butylphthalide | 191.1058;173.0963;128.0598;115.0524;91.0527 | 705798 | *√* | G |
| 53 | 47.67 | [M+H]^+^ |  | 191.1056 | 191.1067 | -5.5 | C_12_H_14_O_2_ | 190.10 | Ligustilide | 191.1066;173.0957;145.0998;115.0525;91.0521 | 4028959 | *√* | G |
| 54 | 48.52 | [M+H]^+^ |  | 233.1533 | 233.1536 | -1.3 | C_15_H_20_O_2_ | 232.15 | Atractylenolide Ⅱ | 233.1528;215.1423;187.1455;177.0901;151.0729 | 190358 | *√* | B |

B: Rhizoma Atractylodis macrocephalae; C: Pericarpium Citri Reticulatae; D: Cortex Moutan; G: Radix Angelicae Sinensis; J: Flos Lonicerae; N: Fructus Ligustri lucidi; Q: Fructus Lycii Barbari; S: Radix Rehmanniae Exsiccata; T: Semen Cuseutae;Semen Cuseutae; X: Radix Scrophulariae; Z : Fructus Corni.
