# Supplementary figures and images for: SIRT1/P53 in retinal pigment epithelial cells in diabetic retinopathy: a gene co-expression analysis and He-Ying-Qing-Re formula treatment
Source: Front Mol Biosci. 2024 Apr 3;11:1366020. doi: 10.3389/fmolb.2024.1366020 (PMC11021775; doi:10.3389/fmolb.2024.1366020)

## Raw data for WB data (in vitro)

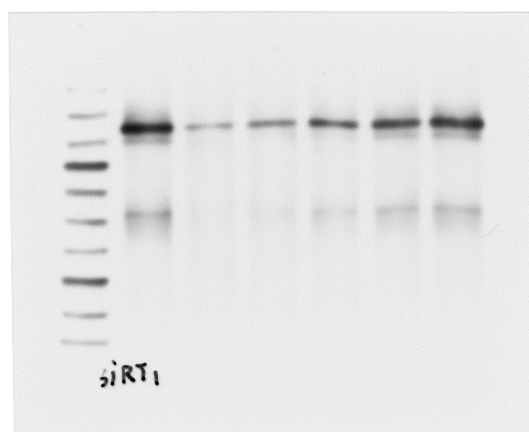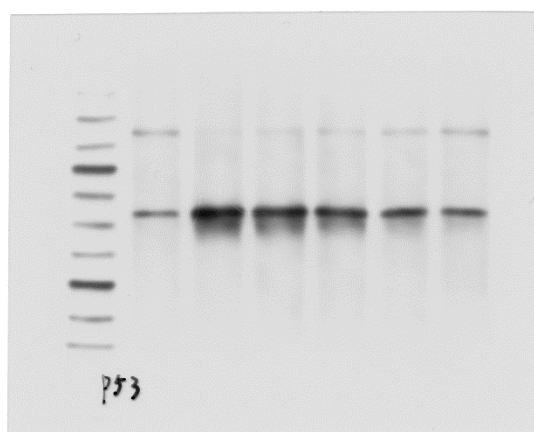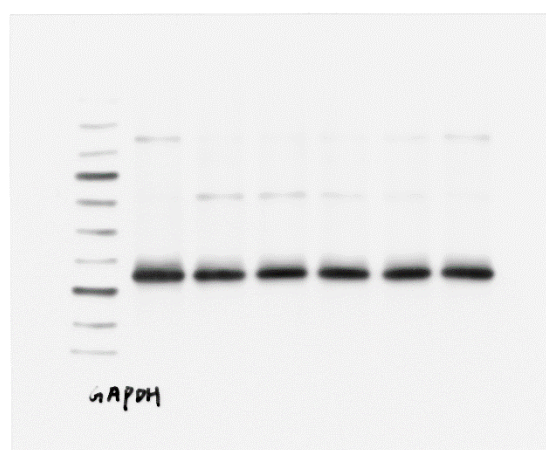

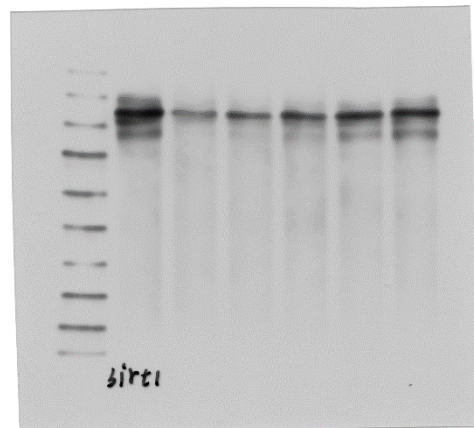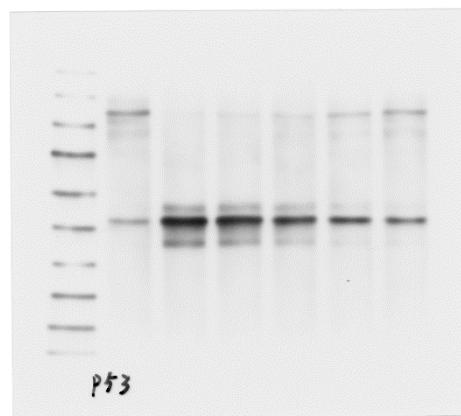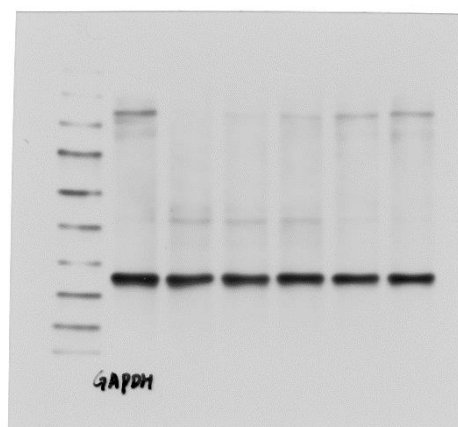

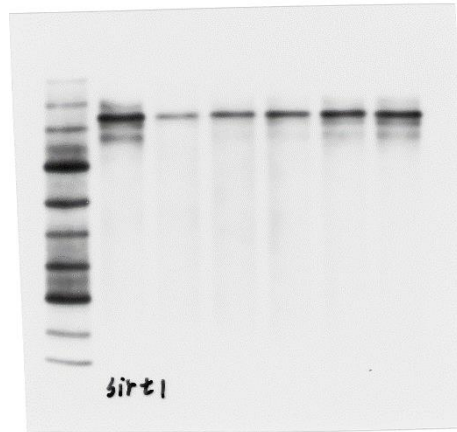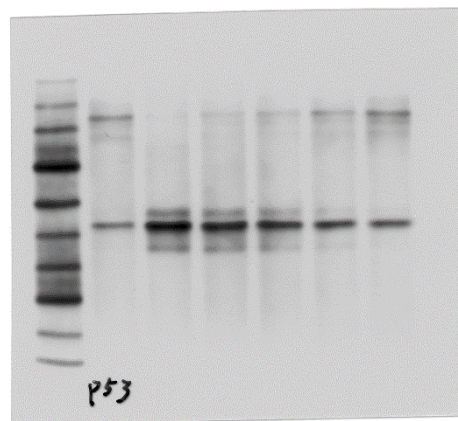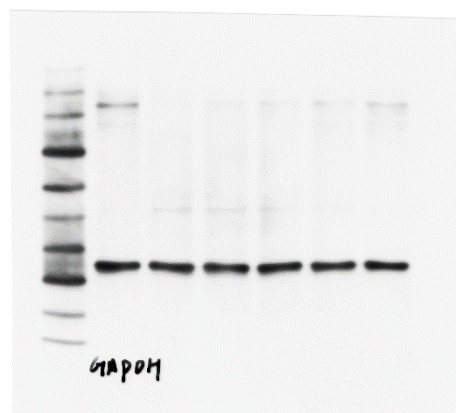

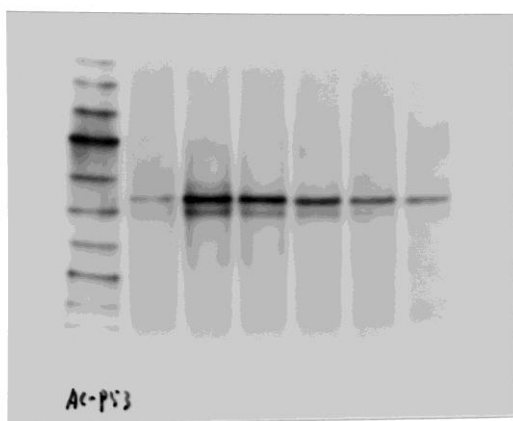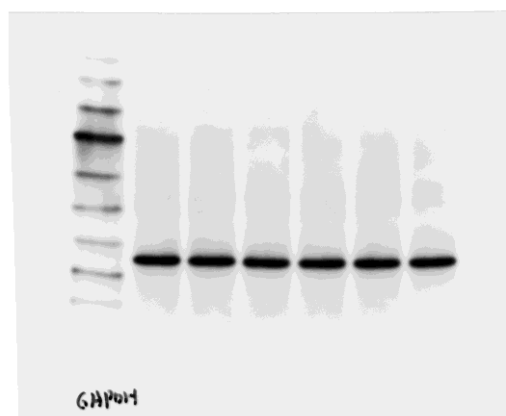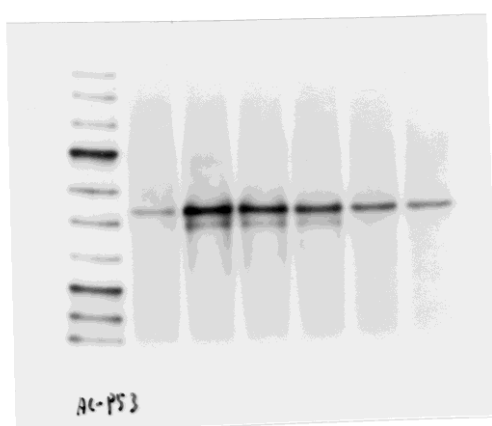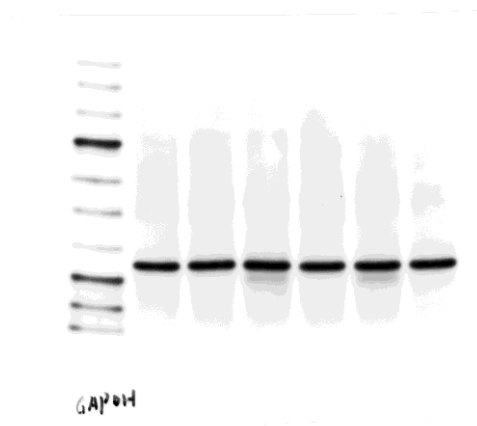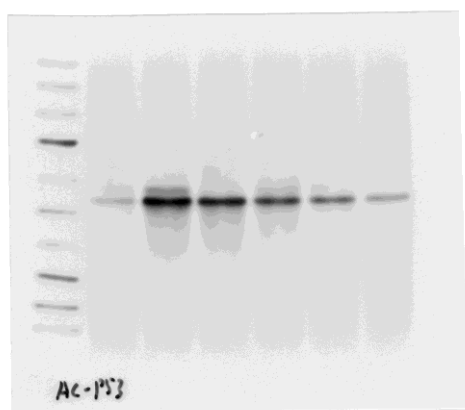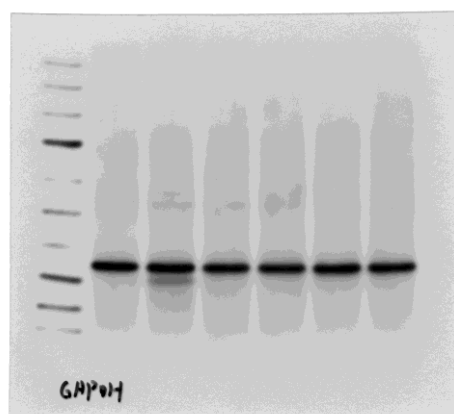

## Raw data for WB data (in vivo)

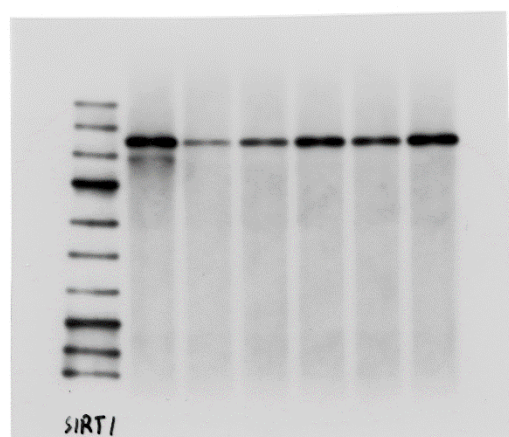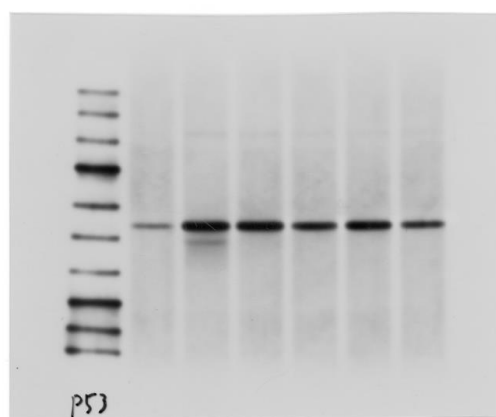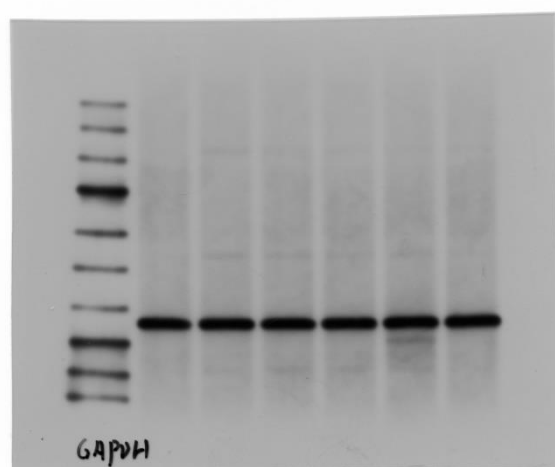

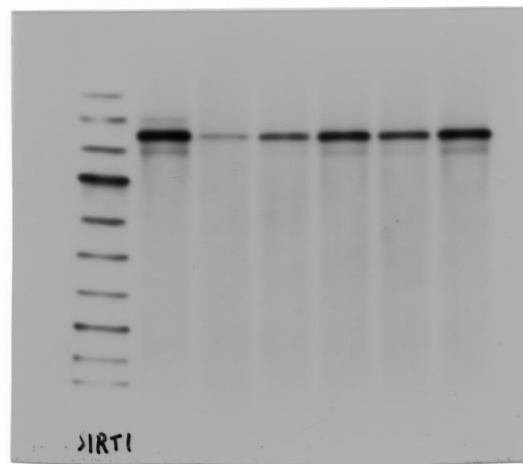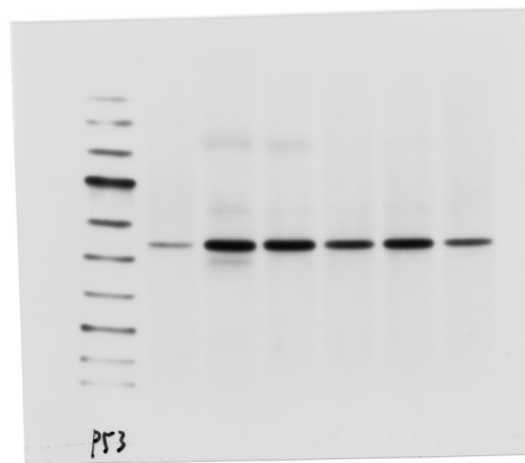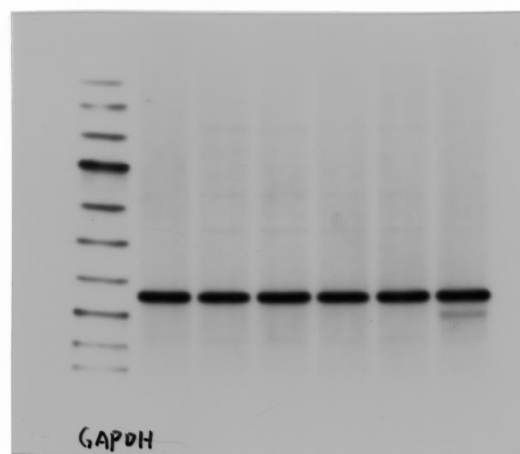

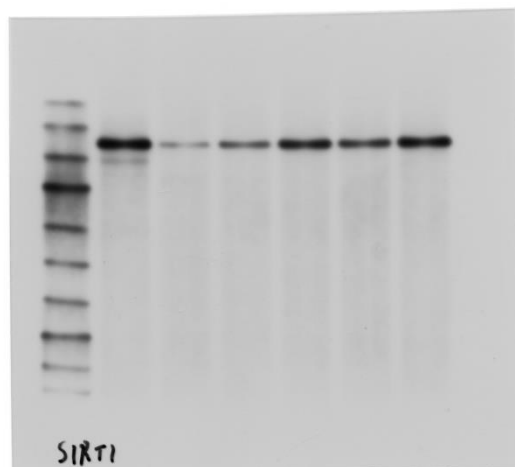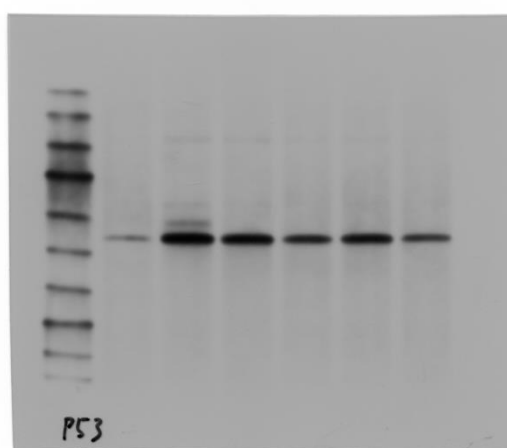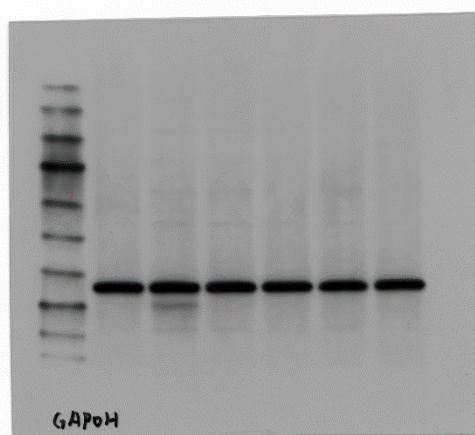

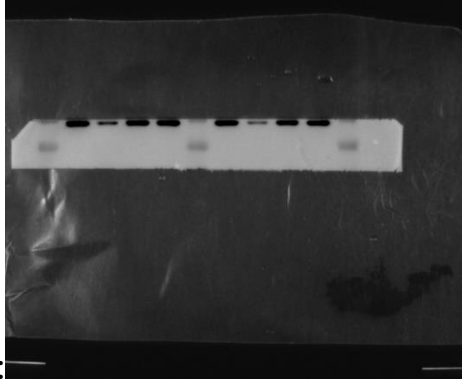

SIRT1:

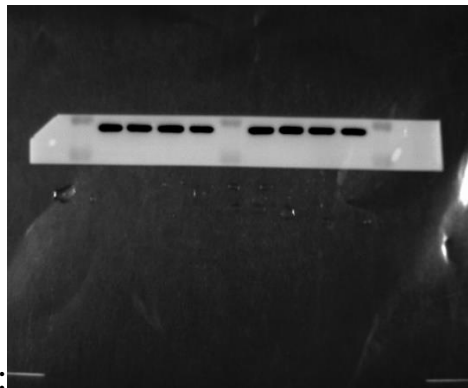

GAPDH:

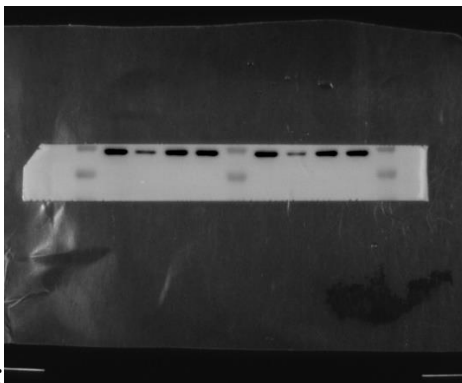

SIRT1:

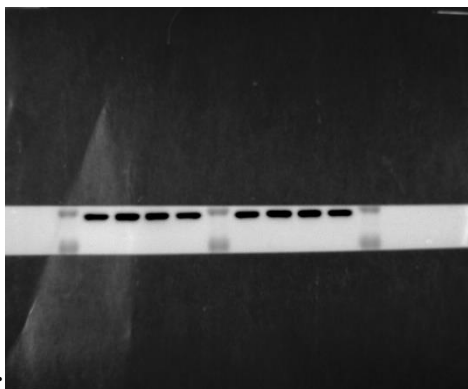

GAPDH:

Supplement: Supplementary file 1 [file DataSheet1.zip › Supplementary Materials/Supplementary Material 6.PDF]
